# Mammalian H4K16ac regulates the spatiotemporal order of genome replication rather than gene expression

**DOI:** 10.1093/nar/gkaf916

**Published:** 2025-09-23

**Authors:** Marta Milan, Valeria Runfola, Manthan Patel, Lucia Falbo, Roberta Noberini, Chiara Soriani, Simona Rodighiero, Tiziana Bonaldi, Vincenzo Costanzo, Madapura M Pradeepa, Paola Scaffidi

**Affiliations:** Cancer Epigenetics Laboratory, The Francis Crick Institute, London, NW1 1AT, United Kingdom; Cancer Epigenetics, Department of Experimental Oncology, IEO, European Institute of Oncology IRCCS, Milan, 20139, Italy; Blizard Institute, Centre for Epigenetics, Faculty of Medicine and Dentistry, Queen Mary University of London, London, E1 2AT, United Kingdom; DNA Metabolism, IFOM-ETS, The AIRC Institute of Molecular Oncology, Milan, 20139, Italy; Department of Oncology and Haemato-Oncology, University of Milano, Milan, 20122, Italy; Nuclear Proteomics, Department of Experimental Oncology, IEO, European Institute of Oncology IRCCS, Milan, 20139, Italy; Imaging Unit, Department of Experimental Oncology, IEO, European Institute of Oncology IRCCS, Milan, 20139, Italy; Imaging Unit, Department of Experimental Oncology, IEO, European Institute of Oncology IRCCS, Milan, 20139, Italy; Department of Oncology and Haemato-Oncology, University of Milano, Milan, 20122, Italy; Nuclear Proteomics, Department of Experimental Oncology, IEO, European Institute of Oncology IRCCS, Milan, 20139, Italy; DNA Metabolism, IFOM-ETS, The AIRC Institute of Molecular Oncology, Milan, 20139, Italy; Department of Oncology and Haemato-Oncology, University of Milano, Milan, 20122, Italy; Blizard Institute, Centre for Epigenetics, Faculty of Medicine and Dentistry, Queen Mary University of London, London, E1 2AT, United Kingdom; Cancer Epigenetics Laboratory, The Francis Crick Institute, London, NW1 1AT, United Kingdom; Cancer Epigenetics, Department of Experimental Oncology, IEO, European Institute of Oncology IRCCS, Milan, 20139, Italy

## Abstract

Histone acetylation is widely assumed to directly instruct gene activation. Among acetylated residues, H4K16ac is one of the most abundant modifications, conserved across all eukaryotes. Despite its established role in X-chromosome hyperactivation in *Drosophila*, its function in mammalian cells has remained elusive. Here, we show that in human somatic cells, H4K16ac does not substantially affect gene expression, but instead controls the spatiotemporal program of genome replication. By combining a meta-analysis of public datasets and perturbation experiments designed to minimize confounding effects, we found that H4K16ac is neither associated with nor required for transcriptional activity. Rather, H4K16ac depletion resulted in premature replication of heterochromatic regions and widespread alterations in replication timing across the genome. These defects were driven by the aberrant activation of cryptic replication origins at long terminal repeats—repetitive elements typically marked by H4K16ac and whose sequence context resembles that of canonical origins in euchromatic regions. Our findings reveal an unexpected role for one of the most prevalent chromatin modifications and uncover a new regulatory mechanism that safeguards genome replication fidelity.

## Graphical Abstract

**Graphical Abstract:**
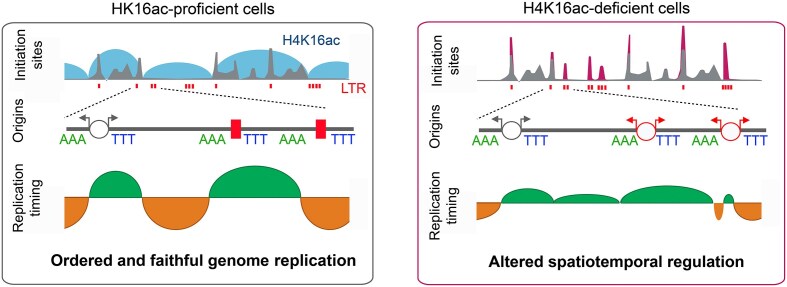

## Introduction

Histone modifications are key components of the epigenome, participating in nearly all nuclear processes within a cell [1]. Among these, histone lysine acetylation is well known for its association with transcriptionally active chromatin. Many residues on both core and linker histones are acetylated and frequently co-enriched at cis-regulatory elements of active genes [2]. Their levels are dynamically regulated by a network of over 30 enzymes, which deposit and remove acetyl groups from histone tails with high turnover rates [3]. Histone acetyltransferases and deacetylases generally exhibit broad substrate specificity, and most individual histone residues are modified by multiple enzymes. For instance, KAT2A modifies H3K9, H3K14, and H3K56; conversely, H3K9 is acetylated by KAT2A, KAT2B, KAT6A, and KAT6B [4]. A notable exception to this is H4K16ac, as it is deposited nonredundantly by one complex, the Male-Specific Lethal (MSL) acetyltransferase complex, which in turn exclusively modifies H4K16 [5, 6]. The mammalian MSL complex consists of three specific subunits (MSL1, MSL2, and MSL3) and the catalytic subunit KAT8, which is shared with the Non-Specific Lethal (NSL) complex [5]. Disruption of the MSL complex via inactivation of MSL-specific subunits results in loss of H4K16ac, with no detectable alterations in other histone marks [5, 7]. Inactivation of KAT8 also affects H4K5ac and H4K8ac due to its role in the NSL complex [5].

H4K16ac is best known for its role in dosage compensation in *Drosophila*, where an enrichment on the male X chromosome drives a two-fold increase in X-linked gene expression to match the levels transcribed in XX females [8, 9]. H4K16ac-driven X chromosome hyperactivation is critical for male fly development, as indicated by penetrant lethality of MSL-defective or H4 mutant males [10]. Despite the evolutionary shift in dosage compensation mechanisms, which in mammals rely on female-specific gene silencing, the H4K16ac mark has been retained in vertebrates, though its physiological role in mammals remains unclear. At the biochemical level, *in vitro* studies have shown that H4K16ac affects inter-nucleosome interactions [11] and reduces chromatin compaction [12]; consistent with these effects, euchromatic domains show a mild enrichment of H4K16ac in human cells [5]. However, perturbation of H4K16ac levels does not affect chromatin accessibility, as assessed by ATAC-seq [5], nor higher-order chromatin compaction [13], complicating the interpretation of the *in vitro* observations. Based on the role of H4K16ac in *Drosophila*, and the broad association of acetylated marks with gene activity, potential links between the modification and transcription have been investigated: while correlations between H4K16ac and transcriptional activity have been described in some contexts, these patterns appear cell type-specific, and a causal relationship has not been established [13, 14]. Deletion of *MSL2* in mice influences biallelic gene expression, causing some genes to shift to monoallelic expression [15]. However, this phenotype is H4K16ac-independent, excluding a role for H4K16ac at this level of transcriptional regulation. More recently, H4K16ac has been shown to mark a subset of transcribed LINE transposable elements (TEs), and depletion of the modification in embryonic stem cells led to a downregulation of these enhancer-like elements, with a mild effect on gene expression [16]. While enrichment of H4K16ac at TEs was also detected in cancer cell lines, whether this is a widespread phenomenon, especially in noncancerous somatic cells, remains to be investigated. Thus, evidence supporting a causal role for mammalian H4K16ac in mediating transcriptional activation is scarce and inconclusive.

Beyond transcription, H4K16ac has been implicated in genome maintenance. Using an unbiased approach, we have recently identified the MSL complex as a vulnerability of genetically unstable cancers [7]: disruption of MSL in growing tumors impaired tumor maintenance in xenograft models of various cancer types, and we have shown that this effect is driven by an extreme chromosomal instability (CIN), which progressively exhausts cancer cells’ proliferative capacity. Interestingly, normal cells can tolerate MSL disruption, making the complex a cancer-specific vulnerability. Initial characterization of the mechanistic basis of this specificity revealed that pre-existing genomic instability sensitizes cells to MSL disruption: loss of H4K16ac induces replication stress that synergizes with oncogene-induced replication defects, ultimately resulting in mis-segregation of abnormally replicated chromosomes [7]. Supporting the notion that H4K16ac may play a role in DNA replication, in human cells its levels peak in S-phase [17] and in yeast the mark is deposited on chromatin immediately upon DNA replication [18]. Furthermore, a link between KAT8 depletion and the response to replication stress induced by treatment with DNA damaging agents has been proposed [19]. However, a MSL-specific effect has not been assessed, making it difficult to directly attribute this function to H4K16ac. Lastly, phosphorylated SIRT1, the main histone deacetylase responsible for removing H4K16ac, has been shown to inhibit replication origin overactivation [20], although the potential role of H4K16ac in this process remains unclear.

**Figure 1.**
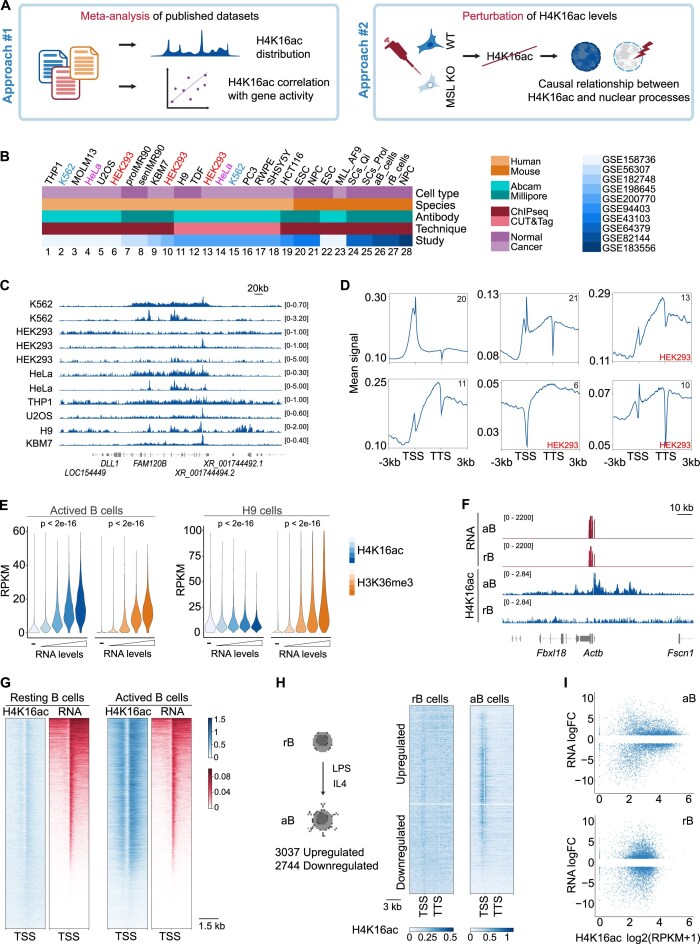
Meta-analysis of H4K16ac distribution and relationship with gene activity in public datasets. (**A**) Schematic of the strategy used in this study. Created in BioRender. Milan, M. (2025), https://BioRender.com/d1vcdqf. (**B**) Summary of public H4K16ac profiling datasets analyzed in this study. Cell lines profiled in multiple studies are color-coded. (**C**) H4K16ac tracks showing high variability in distribution among datasets. (**D**). H4K16ac metaprofiles at coding genes from different datasets. Dataset number in the upper right corner: same as panel (B). TTS, transcription termination site. (**E**) Distribution of ChIP-seq signal at quartiles of gene expression in the indicated cell lines. Reads per kilobase per million (RPKM) are the sum of signal from promoter [1 kb upstream of transcription start site (TSS)] and gene body. *P*-values from one-way Analysis of Variance (ANOVA). (**F**) Tracks showing high level of expression of the housekeeping β-actin gene in both activated (aB) and resting (rB) B cells but lack of H4K16ac enrichment in rB. (**G**) Visualization of H4K16ac and RNA signal at promoter of coding genes in the indicated cell lines. (**H**) Visualization of H4K16ac signal at differentially expressed genes (DEGs; False discovery rate (FDR) < 0.01, |log_2_FC| ≥ 1) in the transition from resting (rB) to activated (aB) B cells, sorted by fold change. (**I**) Relationship between H4K16ac signal (RPKM at promoter—1 kb upstream of TSS—and gene body) and fold change of DEGs. Each dot is a gene. *N* = 5781.

In this study, we employed a dual approach to uncover the primary function of H4K16ac in human, disease-free somatic cells (Fig. 1A). Through a meta-analysis of public genome-wide datasets and perturbation experiments in a controlled cellular model, we set out to reconcile conflicting data from the literature, and establish causal relationships between H4K16ac distribution, transcriptional regulation, and the DNA replication program.

## Materials and methods

### Cell culture

hTert-HME1 cells (ME16C, American Type Culture Collection CRL-4010) were grown in Dulbecco’s modified Eagle’s medium/F12 (Thermo Fisher, 11330057) with the addition of 2% fetal bovine serum (Gibco 16000-044), 100 U/ml penicillin and 100 μg/ml streptomycin (Thermo Fisher, 15140122), 20 ng/ml EGF (Merck, E9644), 0.5 μg/ml Hydrocortisone (Merck, H0888), and 10 μg/ml recombinant human Insulin (Merck, I9278). Cells were maintained at 37°C in 5% CO_2_. To enable efficient CRISPR-induced knock-out (KO), a previously generated clone expressing inducible Cas9 [7] was used throughout the whole study.

### Cell line generation

KO lines were generated either by isolating clonal populations after transducing Cas9-expressing cells with lentiviral constructs expressing MSL-targeting single-guide (sgRNAs) [21] or by transfecting synthetic guide RNAs [Edit-R CRISPR RNA (crRNA), Horizon]. In both cases, guide RNAs targeting the nonexpressed gene *TNP2* were used as negative control, with the exception of experiments scoring pRPA/γH2AX foci, where GFP-targeting sgRNAs were used as a control. For KO cell line generation, pLenti-sgRNA constructs were generated as previously described [21]. Lentiviral particles were produced by transfecting HEK293T packaging cells at 80% confluence in six-well plates with 1 μg of the sgRNA lentiviral construct, 0.75 μg of psPax2 packaging plasmid (Addgene, #12260) and 0.25 μg of pMD2G envelope plasmid (Addgene, #12259) at a ratio of 3:1 FugeneHD to DNA (Promega, #E2311). Forty-eight hours after transfection, the supernatant containing viral particles was recovered, filtered through a 0.45 μm filter (Millipore, #MSHVS4510), diluted 1:4, supplemented with 5 μg/ml polybrene (Santa Cruz, sc-134220), and added to HME1 cells. After selection with 6 μg/ml Blasticidin (S, Calbiochem, 203350), and Cas9 induction with 1 μg/ml doxycycline (Sigma, D9891), KO polyclonal populations were obtained. Single cell clones from MSL3-targeted cells were screened for loss of H4K16ac signal to isolate pure populations of KO cells completely lacking the histone mark.

For acute knock-out experiments, HME1 cells were pre-induced with 1 μg/ml doxycycline 24 h before transfection. For transfection of synthetic guide RNAs, individual crRNA targeting MSL subunits or TNP2 and the *trans*-activating CRISPR RNA (tracrRNA; Horizon, U-002005) were resuspended in 1 × small interfering RNA (siRNA) buffer (Horizon, B-002000-UB-100) at a concentration of 20 μM. Each crRNA was then mixed with an equal amount of tracrRNA and the mix diluted 1:100 in Optimem medium (Gibco, #31985047). A total of 300 μl of the crRNA/tracrRNA mix was added to a well of a six-well plate (to achieve a final concentration of 20 nM in a 2 ml of final volume) and mixed with 300 μl of Optimem containing 4.5 μl of DharmaFECT 4 (Horizon, T-2004-01. After 15 min, 120 000 cells resuspended in 2.4 ml of complete medium containing 1 μg/ml doxycycline were added to each well. All sgRNA sequences are listed in Supplementary Table S1.

For c-MYC overexpression, c-MYC was amplified from complementary DNA (cDNA) of HME1 cells and cloned in pLentiGS-minCMV-TET-blasticidin [22] using the restriction enzymes NheI and NotI. After validation of the construct by Sanger sequencing, MSL3- and TNP2-KO cells were infected with pLentiGS-minCMV-TET-blasticidin or pLentiGS-minCMV-TET- c-MYC -blasticidin with the same protocol described above. To avoid excessive c-MYC overexpression, we exploited the vector leakiness, which increased c-MYC levels by four-fold over the endogenous ones in the absence of doxycycline.

For the generation of stable cell lines expressing the cell cycle reporter FUCCI(CA)2, the lentiviral construct tFucci(CA)2/pCSII-EF, provided by the RIKEN BRC through the National BioResource Project of the MEXT, Japan (cat #RDB15446) was used to produce lentiviral particles as described above and transduce *MSL3*-KO and *TNP2*-KO HME1 cells.

### Proliferation and clonogenic assay

For proliferation assays, cells were seeded in 12-well plates at a confluence of 25 × 10^3^ cells per well, in four replicates. Brightfield images were automatically acquired by Incucyte SX5 Live-Cell System every 6 h for 72 h, at 10× magnification, to monitor cell proliferation. Population growth was performed using IncuCyte 2022B Rev2 Software and plotted as cell confluence over time.

For 2D limiting dilution clonogenic assays, also suitable for nontransformed cells, a matrix of decreasing numbers of cells was seeded in 96-well plates. Starting from 500 cells transferred in the A1 well in 200 μl and through two-fold serial dilutions of cells from top to bottom rows and from left to right columns, the final estimated number of cells seeded in 200 μl of medium in each well ranged from 500 (A1) to 0.002 (H12), with diagonal wells containing identical estimated numbers of cells. Cells were plated in three 96-well plates per condition. At 16 days after plating, whole wells were imaged using an IncuCyte Zoom live cell imager (Essen BioScience) with a × 4 objective, and populated wells containing at least 1 clone of >20 cells were scored manually.

### Aphidicolin sensitivity assay

Three thousand cells were plated in triplicate in 24-well plates, treated with aphidicolin (Sigma, A4487-1ML) for 16 h, and then cultured for 5 days. Five days after plating, cells were fixed with 4% paraformaldehyde (PFA) in phosphate buffered saline (PBS), permeabilized with 0.5% Triton in PBS and stained with SYTOX Green nucleic acid stain (Thermo Fisher, S7020). After a wash in PBS, whole wells were imaged using an IncuCyte Zoom live cell imager (Essen BioScience) with a × 4 objective. Nuclei were quantified using IncuCyte software (v.2020B).

### Immunofluorescence microscopy

Cells were grown on imaging plates (Miltenyi Biotec, 130-098-264) and fixed in 4% PFA (Alfa Aesar, 43368.9) in PBS. Immunostaining was performed following standard protocols using the following primary antibodies: anti-H4K16ac (Cell Signaling Technology, 13534; 1:500); anti-γH2A.X (Millipore, 05636; 1:1000); anti-pRPA S33 (Bethyl Laboratories, A300-246A; 1:200). Secondary antibodies, used at a 1:400 dilution, were as follows: Alexa Fluor 568 donkey anti-rabbit (Thermo Fisher Scientific, A10042); Alexa Fluor 488 donkey anti-mouse (Thermo Fisher Scientific, A21202); and Alexa Fluor 488 donkey anti-rabbit (Thermo Fisher Scientific, A-21206). DNA was stained using 4′,6-diamidino-2-phenylindole (DAPI). Cells were imaged using a Nikon CSU-W1 Spinning Disk or a Nikon LTTL system. For high-throughput imaging and quantification of pRPA/γH2A.X foci, cells were imaged using an Opera Phenix High Content Screening System (PerkinElmer) and quantification was performed using Harmony software as previously described [7].

### Analysis of S phase kinetics by FUCCI(CA)2 reporter

FUCCI(CA)2-expressing MSL3-KO and Cntr-KO HME1 lines were seeded in 96-well plate at a confluence of 2 × 10^3^ cells per well, in six replicates, and treated for 24 h with 0.2 μM Palbociclib (MERCK, #PZ0383) or with PBS, as a control. Twenty-four hours after washout of Palbociclib (multiple washes with PBS), cells were automatically imaged by Incucyte SX5 Live-Cell System, at 10×/0.3NA magnification, to monitor S-phase length. Phase, Green and Orange fluorescence channels were acquired, at 30-min intervals, taking four fields of view per well, for a total duration of 3 days and 2 h. Image and data analysis were carried out adapting an automated pipeline and a custom machine learning algorithm, previously described [23]. Briefly, 16-bit original images of Green and Orange channels exported from Incucyte as TIFF were processed in Fiji (v. 2.16.0/1.54p) [24]: images of Green and Orange channels were imported and a background subtraction was performed; the single channels were then merged in a unique hyperstack and subsequently processed to obtain the Brightness scale, used as tracking channel reference, and the Hue scale, for cell-cycle phase measurement; the Brightness channel was processed with a Top Hat filter (radius = 50 px) for particle enhancement, and the Hue channel was processed with a Remove Outliers function (radius = 3, threshold = 20) to discard hot pixels; the time-lapse hyperstack resulting from the merge of processed Green, Orange, Brightness, and Hue channel was aligned with the *Linear Stack Alignment with SIFT Multichannel* Fiji plugin, to correct for stage drift (https://imagej.net/plugins/linear-stack-alignment-with-sift) [25]. Processed images were then analyzed with a script executing TrackMate plugin [26], using the Laplacian of Gaussian detector as the cell identifier on the Brightness channel, while the Linear Assignment Problem Tracker algorithm was utilized for linking phase, with gap closing active for up to two frames; resulting tracks were filtered in order to discard tracks starting after 10 frames (5 h) and only tracks with a total duration above 35 frames (17.5 h) were kept. The output tables of Spots were subsequently analyzed with the R pipeline as described [23]: a new model for cycling tracks recognition on our dataset was trained, quality checked and subsequently used to select tracks that were completing at least one cycle; these tracks were used for phase assignment and quantification of the relative duration, keeping track of the cycle number (88 < *N* < 560). Single-cell S-phase length data from the second cell cycle analyzed by the algorithm were plotted in bar graphs, for each genotype and condition, ranked from highest to lowest.

### Profiling of histone marks by ChIP-qPCR and ChIP-seq

ChIP-qPCR and ChIP-seq were carried out as previously described [27]. H4K16ac, H3K27me3, and H3K9me3 ChIP were performed in duplicate in monoclonal Ctrl-KO and MSL3-KO HME1 populations while H3K36me3 was performed in HME1 parental cells. A total of 2 × 10^6^ cells per pull down were cross-linked using 1% formaldehyde for 10 min. The reaction was stopped by incubation for 5 min with 0.125 M glycine. After nuclear extraction and chromatin shearing by sonication, lysates were incubated overnight at 4°C with protein G Dynabeads (Thermo Fisher Scientific, 10003D) coupled with one of the following antibodies: anti-H4K16ac (Millipore, 07-329, 5 μg), anti-H4K16ac (Cell Signaling Technology, 13534; 1 μg), anti-H4K16ac (Abcam, ab109463, 5 μg), anti-H3K36me3 (Cell Signaling Technology, 4909S, 0.5 μg), anti-H3K27me3 (Millipore, 07-449, 5 μg), anti-H3K9me3 (Abcam, ab176916, 5 μg), IgG (Abcam, ab46540, 5 μg). After immunoprecipitation, beads were recovered using a magnet and washed; chromatin was recovered in elution buffer (TE containing 2% sodium dodecyl sulphate (SDS)] and cross-links reverted overnight at 65°C. DNA was purified with solid-phase reversible immobilization (SPRI) beads (Agencourt AMPure XP, Beckman Coulter), and then quantified with QuantiFluor (Promega, E2670). For ChIP-seq, DNA libraries were prepared using the NEBNext UltraII DNA Library Prep Kit for Illumina (New England Biolabs, E7645L). Library quality was assessed using TapeStation and running samples on a High Sensitivity D5000 ScreenTape (Agilent, 5067-5592). Samples were pair-end sequenced on an Illumina NovaSeq6000. For H4K16ac pull-downs, Drosophila spike-in was added to normalize signal between Ctrl-KO and MSL3-KO cells: S2 Drosophila cells were processed as described above and lysates quantified using DC Protein Assay Kit I (Bio-Rad Laboratories Ltd, 5000111). 5% chromatin was added to HME1 lysates and pull-down was carried on. For ChIP-qPCR, eluted genomic DNA was diluted 1/10 and used as input for quantitative reverse transcription PCR (RT-qPCR) using SsoAdvanced Universal SYBR Green Supermix (Bio-Rad, 172-5274) on a QuantStudio 7 Real-Time polymerase chain reaction (PCR) System (Applied Biosystems). Enrichment over the input was calculated and expressed as relative to an active promoter (*ATP5F1B* promoter). Primers used for ChIP-qPCR in this study are listed in Supplementary Table S2.

### Profiling of H4K16ac by CUT&Tag

We performed two biological replicates of CUT&Tag on HME1 cells as previously described [28] with a few modifications to sample preparation. Cells were pelleted by centrifugation for 3 min at 600 × *g* at room temperature and resuspended in 500 μl of ice-cold NE1 buffer [20 mM HEPES–KOH, pH 7.9, 10 mM KCl, 0.5 mM spermidine, 1% Triton-X 100, and 20% glycerol and cOmplete ethylenediaminetetraacetic acid (EDTA)-free protease inhibitor tablet (Roche, 11836170001)] and was let to sit for 10 min on ice followed by nuclei preparation. Nuclei were pelleted by centrifugation for 4 min 1300 × *g* at 4°C and resuspended in 500 μl of wash buffer and by placing the tubes on a magnet stand to clear and withdraw the liquid, then resuspended in 1.0 ml wash buffer and held on ice until beads were ready. For each tube, 10 μl of BioMag Plus Concanavalin-A-conjugated magnetic beads (ConA beads, Polysciences, Inc) were resuspended in the binding buffer and kept on a nutator for 10 min. After a pulse spin to remove liquid from the cap, tubes were placed on a magnet stand to clear, the liquid was withdrawn, and 1 μl of primary antibody added—anti-H4K16ac (Abcam, ab109463) or IgG (normal rabbit IgG, Santa Cruz, sc-2027)—resuspended in 800 μl of antibody buffer. After an overnight incubation at 4°C on a nutator, secondary antibodies (guinea pig α-rabbit antibody, Antibodies online cat. no. ABIN101961) were added 1:100 in Dig-wash buffer (5% digitonin in wash buffer) and squirt in 100 μl per sample while gently vortexing to allow the solution to dislodge the beads from the sides and incubated for 60 min on a nutator. Any unbound antibodies were removed by three washes with 1 ml of dig-wash buffer. The complex was then incubated with 100 μl of (1:250 diluted) protein-A-Tn5 loaded with adapters in Dig-300 buffer (20 mM HEPES, pH 7.5, 300 mM NaCl, 0.5 mM spermidine with Roche cOmplete EDTA-free protease inhibitor) and placed on a nutator for 1 h followed by three washes with 1 ml of Dig-300 buffer to remove unbound pA-Tn5. Tagmentation was carried out by adding 300 μl Tagmentation buffer (Dig-300 buffer + 5 mM MgCl_2_) gently while mild vortexing and incubating for 1 h at 37°C. Tagmentation was stopped by adding 10 μl 0.5 M EDTA, 3 μl 10% SDS and 2.5 μl 20 mg/ml Proteinase K to each sample. Samples were mixed by full-speed vortexing for ∼2 s and incubated for 1 h at 55°C to digest proteins. DNA was extracted by phenol: chloroform protocol using phase-lock tubes (Quanta Bio) followed by ethanol precipitation. Libraries were prepared using NEBNext HiFi 2× PCR Master mix (Cat number M0541S) with 72°C gap filling step followed by 13 cycles of PCR with 10-s combined annealing and extension for enrichment of short DNA fragments. Libraries were sequenced in Novaseq 6000 (Novogene) with 150bp paired-end reads at Novogene sequencing service. Tn5 was purified and loaded with the adapters as described in Kaya-Okur *et al.* [28].

### Mapping of nascent DNA by EdU-HU-seq

HME1 cells of the analyzed genotypes were seeded in T150 flasks. When 50% confluency was reached, cells were treated with 2 μM CDK4/6 inhibitor Palbociclib (Universal Biologicals, S116) for 20 h to synchronize them in G1/S. Cells were washed twice with medium and treated with 10 mM Hydroxyurea (Merck Life Science, H8627) for 7 h to arrest firing replication origins in early S phase. After two washes with medium, cells were released in medium containing 25 μM EdU (Universal Biologicals, 10540) for 90 min, a time that we found enabled efficient pull-down of nascent DNA (nDNA), while maintaining high-resolution mapping of initiation sites. Cells were collected and fixed in 90% MetOH. EdU-labeled DNA was isolated as previously described [29]. Briefly, cells were permeabilized in 0.2% Triton-X 100 in PBS for 30 min and EdU was detected using a biotin-azide click reaction by incubating cells in click-it reaction cocktail [100 mM Tris–HCl, pH 8, 4 mM CuSO_4_, 100 mM sodium-l-ascorbate, 50 μM Biotin Azide (Thermo Fisher Scientific, B10184)] for 30 min. After washes, cells were incubated in lysis buffer (10 mM Tris–HCl, pH 8, 10 mM EDTA, 0.5% SDS, 0.2 mg/ml Proteinase K) for 3 h at 50°C. DNA was purified using phenol/chloroform/isoamyl alcohol, resuspended in TE and sonicated using Bioruptor Pico. EdU-labeled DNA was captured using Dynabeads MyOne Streptavidin C1 (Thermo Fisher Scientific, 65001), washed and eluted from beads using 2% β-mercaptoethanol. DNA was purified with SPRI beads (Agencourt AMPure XP, Beckman Coulter), and then quantified with QuantiFluor (Promega, E2670). DNA libraries were prepared using the NEBNext UltraII DNA Library Prep Kit for Illumina (New England Biolabs, E7645L). Library quality was assessed using TapeStation and running samples on a High Sensitivity D5000 ScreenTape (Agilent, 5067-5592). Samples were pair-end sequenced on an Illumina NovaSeq6000.

### Definition of firing DNA replication origins by NocoEdU-HU-seq

Ctrl-KO HME1 cells were seeded in T150 flasks. When 50% confluency was reached, cells were treated with 100 ng/ml Nocodazole (Merck Life Science, SML1665) for 8 h. Cells in mitosis were detached by shake-off in the same culture medium, pelleted, washed once with PBS and plated in medium containing 25 μM EdU (Universal Biologicals, 10540) and 10 mM Hydroxyurea (Merck Life Science, H8627). After 16 h, cells were collected and fixed in 90% MetOH. EdU-labeled DNA isolation and library preparation were carried out as described for EdU-HUseq.

### Assessment of DNA replication timing by REPLI-seq

Ctrl-KO and MSL3-KO HME1 cells were seeded in T150 flasks. While in exponential growth phase, asynchronous cells were pulsed with 25 μM EdU (Universal Biologicals, 10540) for 30 min. After fixation with 90% MetOH and permeabilization, Biotin Click it reaction was performed as described above. DNA was stained using PI (propidium iodide). Two populations of cells, corresponding to early and late S phase cells were sorted using Beckman Coulter—MoFlo™ XDP. Cells were then lysed and processed as described for EdU-HU-seq to obtain and sequence EdU-labeled DNA.

### DNA fiber assay

Cells were seeded in a six-well plate, and after 24 h, the cells were labeled with 50 μM 5-chloro-2′-deoxyuridine (CldU, Sigma–Aldrich, C6891) for 30 min. The cells were then washed twice with warm PBS and labeled with 250 μM 5-iodo-2′-deoxyuridine (IdU, Sigma–Aldrich, I7125) for an additional 30 min. After trypsinization, cells were counted and resuspended in PBS to a final concentration of 1–2 × 10^3^ nuclei/ml. Two microliters of the cell suspension were lysed on a clean glass slide with 8 μl of MES lysis buffer (50 mM MES pH 5.5, 0.5% SDS, 50 mM EDTA) for 6 min. Subsequently, the slide was tilted at a 15-degree angle to allow the DNA to spread. The slides were air-dried for 30 min, fixed in freshly prepared methanol: acetic acid (3:1) for 10 min, and then air-dried and stored at 4°C overnight. For immunodetection, the slides were rehydrated with 1× PBS for 5 min. The DNA was denatured in 2.5 M HCl for 60 min, neutralized with PBS, and then blocked in blocking solution [5% bovine serum albumin (BSA), 0.2% Triton X-100 in PBS] for 1 h at room temperature. Next, the slides were incubated with a primary antibody mix consisting of an anti-BrdU antibody (Ab6326, recognizing CldU) and an anti-BrdU antibody (BD Biosciences, #347580, recognizing IdU) both at a dilution of 1:50, in blocking solution for 2 hrs at 37°C in a humid chamber. After incubation, the slides were quickly washed once with PBS 0.1% Tween and twice with PBS for 3 min each. Subsequently, the slides were incubated with a secondary antibody mix consisting of an F(ab')2-Goat anti-Mouse IgG (H + L) Highly Cross-Adsorbed, Alexa Fluor 568 (1:100, A-11019, Thermo Fisher) and a Chicken anti-Rat IgG (H + L) Cross-Adsorbed Alexa Fluor 488 (1:100, #A-21470; Thermo Fisher) in a blocking solution for 1 h at 37°C in a humid chamber. The slides were then washed three times with PBS, air-dried, mounted in Vectashield plus (Vector Labs), and stored at 4°C until image acquisition. Image acquisition was performed with a fully motorized widefield microscope controlled by MetaMorph (Universal Imaging Corporation), using a 60× oil immersion objective. Approximately 25 images were captured per condition, and at least 150 fibers were measured using ImageJ software (version 2.16.0/1.54p). The experiments were repeated three times in triplicate. Micrometer values were expressed in kilobases using a conversion factor of 1 μm = 2.59 kb.

### RNA-seq

To assess H4K16ac’s impact on gene expression programs, avoiding the activation of potential compensatory mechanisms in isolated clones, acute inactivation of MSL1, MSL2, or MSL3 RNAs was carried out using synthetic guides as described above in six-well format. Three days after transfection, cells were collected, and RNA extracted using a RNeasy Plus Micro Kit (Qiagen, 74034). KO efficiency was probed by immunofluorescence assessing loss of H4K16ac. Ribosomal RNA removal was performed with FastSelect -rRNA HMR Kit (Qiagen, 334386) and libraries were prepared using NEBNext UltraII Directional RNA Library Prep Kit (NEB, E7760).

To assess H4K16ac’s impact on global messenger RNA (mRNA) levels, Ctrl-KO, and MSL3-KO HME1 clones were grown for 2 days in two replicate plates. Cells in one plate were fixed with 4% PFA (Alfa Aesar, #43368) followed by permeabilization with 0.5% Triton X-100 in PBS. After nuclei staining with SYTOX Green Nucleic Acid Stain (Thermo Fisher Scientific, #S7020), accurate quantification of cell number was performed using an Incucyte S3 Live-Cell Analysis System. The second plate was scraped in RNeasy lysis buffer and a volume corresponding to 400.000 cells was processed for RNA extraction using a RNeasy Plus Micro kit (Qiagen, 74034). RNA concentration was quantified with a Nanodrop (Thermo Scientific). One microgram of RNA was taken from the lowest concentrated sample and same volume was taken for all the other samples. Two microliters (1:100) of ERCC Spike-in (Thermo Fisher Scientific, 4456740) was added to each sample to enable comparison of global mRNA levels. Libraries were prepared using NEBNext UltraII Directional RNA Library Prep Kit (NEB, E7760) with polyA enrichment.

### Reverse transcription (RT) and qPCR

RNA was extracted using a RNeasy Plus Micro Kit (Qiagen, 74034) according to the manufacturer’s guidelines. Five hundred nanograms of RNA was reverse transcribed using a High Capacity cDNA Reverse Transcription Kit (Thermo Fisher Scientific, 4368814) as per manufacturer’s instructions. cDNA was diluted 1/10 and used as input for RT-qPCR using SsoAdvanced Universal SYBR Green Supermix (Bio-Rad, 172-5274) on a CFX96 real-time PCR detection system (Bio-Rad). *GAPDH* was used as reference gene for normalization. Primers used for RT-qPCR in this study are listed in Supplementary Table S2.

### Mass spectrometry

Histones were enriched from 1 × 10^6^ cells as previously described [30]. Approximately 4 μg of histone octamer were separated on a 17% sodium dodecyl sulphate–polyacrylamide gel electrophoresis gel, either directly (PXD039819) or after mixing with an equal amount of a super-SILAC internal standard [31] (PXD066155). Histone bands were excised, chemically acylated with propionic anhydride and in-gel digested with trypsin, followed by peptide N-terminal derivatization with phenyl isocyanate [32]. Peptide mixtures were separated by reversed-phase chromatography on an EASY-Spray column (Thermo Fisher Scientific), 25-cm long (inner diameter 75 μm, PepMap C18, 2 μm particles), which was connected online to a Q Exactive Plus or HF instrument (Thermo Fisher Scientific) through an EASY-Spray™ Ion Source (Thermo Fisher Scientific), as described [32]. The RAW data were analyzed using the integrated MaxQuant software v.1.6.2.10, against the Uniprot human proteome UP000005640, downloaded on 15/02, filtered to retain only histone sequences, as described [32]. The acquired RAW data were analyzed using EpiProfile 2.0 [33], selecting the SILAC option, followed by manual quantification for the differentially acetylated forms of histone H4 and the peptide containing modifications on H3K27 and H3K36. Identifications, retention times, and elution patterns were used to guide the manual quantification of modified peptide using the QualBrowser software (version 2.0.7, Thermo Fisher Scientific). A % relative abundance (%RA) value was estimated by dividing the area under the curve of each modified peptide for the sum of the areas corresponding to all the observed forms of that peptide and multiplying by 100. H4K16ac and H3K36me3%RAs were calculated by summing all the peptide forms containing the modification. Although %RAs can be affected by the peptide physicochemical properties, it has been shown that H4K16ac, H3K36me3, and H3K27ac have only mild effects on detection efficiencies [34]. Thus, %RAs can be used as an indication of their real abundance. To evaluate the effect of MSL KO on histone PTM levels (Supplementary Fig. S4D and E) %RAs value for the sample (light channel – L) and the internal standard (heavy channel – H) were used to calculate Light/Heavy (L/H) ratios. The mass spectrometry data have been deposited to the ProteomeXchange Consortium [35] via the PRIDE partner repository with the dataset identifier PXD039819 and PXD066155.

### Quantification and statistical analysis

#### Analysis summary

Experimental sample sizes were based on the variability observed in pilot experiments. The type of statistical tests performed in this study, whether they were one- or two-tailed, the value of N, and what N represents are indicated in the main text or in figure legends. Unless otherwise stated, all values are the average of individual values ± standard deviation (SD) from at least three biological replicates. Statistical analysis was performed using the indicated NGS-related packages or GraphPad software.

Where data are shown as boxplot, whiskers represent the 10th and 90th percentiles and outliers were removed for clearer visualization purposes.

One, two, three, or four asterisks: *P*-value <.05, .01, .001, .0001, respectively.

#### RNA-seq analysis

##### Alignment and basic processing

FASTQ files were processed using the RNA-seq pipeline v3.13.2 available at nf-core [36] against the *Homo sapiens* hg38 genome or the *Mus musculus* mm10 genome with “–aligner star_rsem” parameter. For spike-in normalized experiment, the “–additional_fasta” parameter was used to add ERCC spike-in fasta to the alignment. Generated BigWig and count tables were used for downstream analysis.

##### Differential expression analysis

Differential expression analysis was performed with the DESeq2 package [37] within the R programming environment (version 4.4.0) [38] using the count table generated through nf-core. An FDR ≤ 0.01 and a log_2_ fold change ≥ |1| were used as thresholds for identifying DEGs. For acute inactivation of MSL subunits in HME1s, an additional filter for BaseMean > 5 was used to filter out lowly-expressed genes. For the assessment of global mRNA levels, size factors were calculated on spike-in counts only and applied to the human transcript counts. The resulting normalized counts were used to evaluate possible global changes in MSL KO cells.

#### ChIP-seq analysis

##### Alignment and basic processing

FASTQ files were processed using the ChIP-seq pipeline v2.0.0 available at nf-core [36] against the *H. sapiens* hg38 genome or the *M. musculus* mm10 genome with default broad peak calling with MACS2. Generated Bam and BigWig files were used for downstream analysis. For H4K16ac profiling in HME1 cells, a composite genome containing both *H. sapiens* hg38 and *Drosophila melanogaster* BDGP6 genome assemblies was generated and used for alignment via the ChIP-seq pipeline v2.0.0.

##### H4K16ac signal normalization and correction in HME1 cells

From aligned BAM files, SAMtools [39] “view” was used to extract the number of reads aligning to the Drosophila genome in each sample. DeepTools [40] “bamCoverage” was then used to generate BigWig files with a scaling factor calculated as reads per million (RPM) of Drosophila reads. To obtain spike-in normalized H4K16ac signal, “bigwigCompare” was applied to subtract signal detected in MSL KO cells from signal in control cells with parameters –operation subtract –skipNAs -bs 1 –skipZeroOverZero. The resulting BigWig files were used for visualization of normalized tracks in IGV [41]. For downstream analysis, background correction for Millipore and Cell Signaling Technology antibodies was performed using an alternative strategy to account for the nonspecific peaks detected in MSL KO cells. Starting from BAM files, we calculated a scaling factor based on H4K16ac reads present in the common peaks detected in both control and MSL KO samples: DeepTool “multiBamSummary” was used to compute the number of reads covering the top 10 000 common peaks in each sample. Derived scaling factors were used to generate BigWig files through deepTools “bamCoverage” and wild-type (WT) over MSL KO corrected BigWig files were obtained through “bigwigCompare”, with the same parameters specified above. Since negative signals would not be biologically meaningful, “bigWigToBedGraph” and “bedGraphToBigWig” from UCSC tools were applied to bring negative signals to zero. These steps were performed on individual replicates for both H4K16ac antibodies used. Once reproducibility was positively assessed, replicates were merged using SAMtools “merge” and normalized as described for single replicates and used to generate all the data presented in the study.

##### Profiles and heatmaps

Metaprofiles and heatmaps at genes and TEs were generated using deepTools. The “computeMatrix” was used against the BigWig files for each sample to provide a matrix of the density of ChIP-seq signal; “plotProfile” and “plotHeatmap” were then used to generate graphs in the specified kb window. For repetitive elements, the RepeatMask files for *H. sapiens* hg38 genome and the *M. musculus* mm10 genome were downloaded and bed files for the different classes of elements generated by filtering.

##### Comparison of H4K16ac profiles from public datasets

To compare H4K16ac profiles at different genomic elements, the signal at each bin of the matrix from “computeMatrix” was averaged and scaled for each sample separately. The Spearman correlation among the resulting signal distributions—a measure of profile shape similarity across samples, was calculated and unbiased clustering performed to identify possible variables associated with distinct profile shapes.

##### Association of H4K16ac levels with gene activity or genomic features

To evaluate H4K16ac signal at genes, the matrix generated by deepTools “computeMatrix” was used as a source of ChIP-seq signal data. For public datasets, signal at promoters (1 kb upstream the TSS) and at the gene body was combined to account for differences in signal distribution between datasets. To assess the correlation of H4K16ac and H3K36me3 with gene activity in HME1 cells, RPKM from the gene body were normalized to the median of the higher quartile to enable comparison of the correlation independently of the extent of enrichment at genes for the two histone modifications.

To assess the H4K16ac distribution across HiC-defined chromatin compartments, we used the datasets generated by Rao *et al.* in HMEC cells [42] and Peycheva *et al.* in CH12 cells [43] to define A and B compartments. FASTQ files were processed using the HiC pipeline v2.1.0 available at nf-core [36] against the *H. sapiens* hg38 genome or the *M. musculus* mm10 genome to define A and B compartments. H4K16ac counts (or counts for control histone marks) in each 50 kb bin of the genome were calculated using deepTools “multiBamSummary” and then converted in RPKM. For H4K16ac signal in HME1 cells, deepTools multiBigWigSummary was used to be able to use the corrected signal. Bins were finally split based on their overlap with A or B compartments.

To quantify the fraction of the human genome marked by H4K16ac, deepTools “multiBigWigSummary” was used to calculate signal in each 10 kb genomic bin. A bimodal distribution of H4K16ac signal was obtained and a threshold of positivity set at the lowest frequency value observed between the two subsets of bins.

#### CUT&Tag analysis

##### Alignment and basic processing

FASTQ files were processed using the cutandrun pipeline v3.1 available at nf-core [36] against the *H. sapiens* hg38 genome with parameter –normalisation_mode CPM. Generated BAM and BigWig files were used for downstream analysis following the same pipelines used for ChIPseq data.

##### H4K16ac signal normalization in HME1 cells

Signal normalization was carried out as previously described [16] using “bigwigCompare” with default –operation log_2_. Normalised signal was used to assess signal at coding genes and HiC compartments as described for ChIP-seq. Once reproducibility was positively assessed, replicates were merged using SAMtools “merge” and used to generate the data presented in the study.

#### EdU-HU-seq, OK-seq, NS-seq, SNS-seq, and Ini-seq2 analysis

##### Alignment and basic processing

FASTQ files were processed using the ChIP-seq pipeline v2.0.0 available at nf-core [36] against the *H. sapiens* hg38 genome with default broad peak calling with MACS2. Generated BAM and BigWig files were used for downstream analysis.

##### Definition of DNA replication initiation sites

To define replication initiation sites in the EdU-HU-seq dataset, an approach similar to the one used by Tubbs *et al*. [44] was applied. A comprehensive list was generated by combining peaks called in any sample. Peaks within 10 kb were then merged using bedtools “merge”, and only regions with a minimum size of 20 kb were kept. Visual inspection of IGV tracks confirmed accurate calling of robust initiation sites.

##### Identification of differential replication initiation sites

Identification of activated or deactivated sites was performed with the DiffBind package [45] within the R programming environment (version 4.4.0). The analysis was performed on the comprehensive peak set defined as indicated above. Replication initiation sites were defined as differential if meeting the following criteria: concentration (i.e. peak magnitude) of 7 in at least one condition, log_2_ fold change ≥ |0.7|, FDR ≤ 10^−10^.

##### Profiles and heatmaps

Metaprofiles and heatmaps at initiation sites were generated using deepTools. The “computeMatrix” was used against the BigWig files for each sample to provide a matrix of the density of EdU-HU-seq signal; “plotProfile” and “plotHeatmap” were then used to generate graphs in the specified kb window.

##### Enrichment analysis at replication initiation sites

For evaluation of gene enrichment at replication initiation sites, the central 50 kb of each peak was used to avoid differences in size to affect abundance calculation. Overlapping coding genes were found using bedtools “closest” function and quantified.

To calculate histone modification signal at differential replication initiation sites, the matrix generated by deepTools “computeMatrix” for datasets from this study (H3K36me3), GSE134744 (H3K27ac, HME1 cells), GSE57498 (H3K27me3, HMEC cells), and GSE29611 (H3K9me3, HMEC cells) was used as source of ChIP-seq signal data. Signal at the 200 kb region surrounding the site’s center was integrated and plotted.

To evaluate TEs enrichment, a permutation test was deployed using region [46]. The central 50 kb of each replication initiation site was used to avoid confounding factors and permutation analysis performed to evaluate the enrichment of each class of repetitive element within the RepeatMask table (downloaded from UCSC for hg38) in activated replication origins with parameters evaluate.function = numOverlaps, ntimes = 5000. As background, combined constitutive and deactivated sites were used.

##### Nucleotide content and poly-d(A)/poly-d(T)tracts enrichment at long terminal repeats

Bigwig files with nucleotide percentage over 5 nucleotide bins were generated. Briefly, bedtools “makewindows” was used to divide the genome in 5bp bins. bedtools nuc was then used to calculate the abundance of each single nucleotide in every window and the resulting bed file was used to generate the bedGraph file with the nucleotide percentage in each bin. bedGraph files were then converted in bigwig files via “bedGraphToBigWig” function from UCSC and used to generate profiles at TEs with deeptools functions. Profile of long terminal repeat (LTR) located on the plus strand is shown in the figures to avoid confusion from the mismatch between LTR and sequence directionality. Evaluation of polyA/polyT tracts was performed as previously done [44]. To construct the poly-nucleotide tract bigwig files, SeqIO from Python Bio was used to mark nucleotide when inserted in tracts of at least 20 bp with a minimum percentage of 75% of the same nucleotide. A score of 100% was assigned when the condition was met, 0 otherwise. The bed file was converted to bedGraph file and then to bigwig file using “bedGraphToBigWig” (from UCSC).

##### Histone enrichment analysis at LTRs

To calculate histone modification signal at differential LTRs, the matrix generated by deepTools “computeMatrix” for datasets from this study (H3K36me3), GSE134744 (H3K27ac, HME1 cells), and GSE29611 (H3K9me3, HMEC cells) was used as source of ChIP-seq signal data. Signal at the LTR body and the flanking 1kb region was averaged and plotted.

##### Enrichment analysis at LTRs

Metaprofiles at LTRs were generated using deepTools. The “computeMatrix” was used against the BigWig files for each sample to provide a matrix of the density of signals; “plotProfile” was then used to generate graphs in the specified kb window. All signals were stranded based on LTRs orientation. For Ini-seq2, data for HL and LL fraction were aligned as indicated above and bigwig files obtained as previously described [47]. Briefly, “bigwigCompare” within deepTools was used to generate the log_2_ HL/LL BigWig file for downstream profiling. Default –-operation parameter was applied (log_2_) with –binSize 20.

#### Fork progression analysis based on EdU signal

To estimate the relative efficiency of fork progression across genotypes, the NocoEdU-HU-seq dataset was used to define core replication initiation sites corresponding to clusters of firing origins in control HME1 cells. The summit of NocoEdU-HU peaks was used to center progression of bidirectional replication forks (see Macheret *et al.* [48]). Deeptools “computeMatrix” and “plotProfile” were used to plot EdU-HU signal relative to core initiation sites, with the displacement from the NocoEdU-HU signal being a proxy for fork speed in the different conditions. EdU-HU-seq does not have the resolution to map individual origin, but this approach allows us to estimate the rate of EdU incorporation in the regions flanking the core initiation sites, which are replicated as forks progress after HU release.

#### REPLI-seq analysis

##### Alignment and basic processing

FASTQ files for each sample were processed via the ChIP-seq pipeline v2.0.0 available at nf-core [36] against the *H. sapiens* hg38 genome with default broad peak calling with MACS2. Generated BAM and BigWig files were used for downstream analysis.

##### Generation of replication timing tracks

“bigwigCompare” within DeepTools was used to generate log_2_ Early/Late BigWig files for genome browsing and visualization in IGV. Default –-operation parameter was applied (log_2_) with –binSize 20. Once reproducibility was assessed, replicates were merged using SAMtools “merge” and BigWig files generated using “bamCoverage”. “bigwigCompare” within DeepTools was then used to generate log_2_ Early/Late BigWig files as above.

##### Replication timing differential analysis

Differential analysis was performed as previously described [49], with minor modifications. Briefly, counts from each 50 kb bin in the genome were extracted using “multiBamSummary” from DeepTools. Replication timing index (RTI) was calculated as described, without performing quantile normalization to preserve global differences. Each bin was assigned an RTI spanning from −1 (late) to 1 (early) for each replicate in WT and MSL3 KO cells and differential bins were defined as RTI difference > |0.2| and FDR < 0.01.

To measure the overlap with E/L transition, consecutive differential bins were merged. The RTI from 50 kb upstream, the center of differential region, and 50 bk downstream were compared. Regions with concordant increases or decreases in RTI were identified as part of a transition area.

To evaluate the impact of H4K16ac loss on global replication timing (RT), counts from each 50 kb bin in the genome were extracted using “multiBamSummary” from DeepTools. Log_2_ ratios were calculated, and the resulting RT value in control or MSL KO cells was plotted after filtering for regions without reads.

##### Repetitive element enrichment analysis

Permutation analysis was performed using regionR [46] to evaluate the enrichment of each class of repetitive element within the RepeatMask table (downloaded from UCSC for hg38) in anticipated regions with parameters evaluate.function = numOverlaps, ntimes = 5000. The rest of the genome was used as background. To quantify RT at TEs, in each sample was calculated. For regional analysis, the log_2_ ratio of early and late counts in the 50 kb regions surrounding each LTR was used, while only counts covering the elements were used for local analysis. In both cases, counts were extracted using “multiBamSummary” from DeepTools.

## Results

### Inconsistent H4K16ac distribution and association with transcriptional activity in public datasets

Acetylation of different residues on H3 and H4 is generally thought to serve similar functions and promote gene transcription by establishing a chromatin environment conducive to transcription, particularly at promoters and enhancers [4]. Circumstantial evidence suggests that H4K16ac may function differently [7, 13, 16, 50], but the absence of a systematic analysis leaves its potential divergence from other acetylation marks unclear. To gain insights, we began by characterizing H4K16ac genome-wide distribution across diverse cellular models (Fig. 1A, left). We collected 28 public H4K16ac datasets that profiled various human or mouse cell types, using multiple profiling methods and antibodies, across different research laboratories (Fig. 1B and Supplementary Table S3). All datasets were processed from the raw FASTQ files and analyzed using a standardized pipeline to ensure comparable outputs (Supplementary Fig. S1A). Unlike other acetylated histone marks, which are strongly enriched at active gene promoters, clear H4K16ac peaks were only observed in a few datasets, and enrichment patterns were highly inconsistent (Fig. 1C and Supplementary Fig. S1B). H4K16ac signal at genes was variable even across datasets profiling the same cell line (Fig. 1C and D). Inconsistencies were particularly pronounced when comparing datasets generated via ChIP-seq and CUT&Tag or using different antibodies, suggesting that technical aspects may contribute to the observed differences (Supplementary Fig. S1B and E).

Inconsistent patterns extended to the association with gene transcriptional activity. For instance, while a correlation was observed in Lipopolysaccharide (LPS)-activated mouse B cells, with higher H4K16ac levels at highly transcribed genes similarly to H3K36me3 patterns, the mark was present at comparable levels at lowly and highly transcribed loci in human H9 embryonic stem cells (Fig. 1E). Moreover, B cells exhibited H4K16ac peaks at genes only after activation, while in resting cells, even highly expressed housekeeping genes such as β-actin were not decorated by the mark (Fig. 1F). The uncoupling between H4K16ac and nascent RNA levels was evident across the whole transcriptome, demonstrating that steady-state gene activity is independent of H4K16ac levels even in cell types where a context-specific association can be detected (Fig. 1G).

Furthermore, H4K16ac levels did not correlate with dynamic transcriptional activity: the transition from resting to activated B cells is accompanied by expression changes in nearly 6000 genes, yet H4K16ac signal did not show consistent changes (Fig. 1H and I). Similar results were observed in other datasets examining the transitions between quiescent and proliferating muscle satellite cells, and between embryonic stem cells and neuroprogenitor cells (Supplementary Fig. S1F–H).

We conclude that H4K16ac profiles at genes are dataset-specific and likely confounded by technical variability across studies and biological contexts. Steady-state gene activity, as well as dynamic expression changes, are independent of H4K16ac enrichment at genes, suggesting a limited involvement of the mark in the process.

### H4K16ac distribution at other genomic features across datasets

Beyond genes, assessment of H4K16ac distribution at different scales has recently linked the mark to other genomic features. At a larger scale, H4K16ac has been shown to be enriched in euchromatic, gene-dense A compartments defined by conformation capture technologies [5]. Confirming those observations, we found a consistent enrichment of H4K16ac in A compartments compared to B compartments across the 28 datasets, independently of the profiling method and the cell type, showing similar patterns as the active mark H3K27ac and opposite ones compared to the heterochromatin mark H3K9me3 (Supplementary Fig. S1C and D). However, control pull-downs employing IgG showed a similar enrichment in A compartments, questioning the reliability of the detected patterns (Supplementary Fig. S1D). At a smaller scale, H4K16ac has been reported to be enriched at subsets of TEs, including SINEs, LTRs, and the LINE subclass of full-length L1 elements [16]. Across the analyzed datasets, CUT&Tag consistently detected enrichment at the three TE groups, while ChIP-seq profiles were variable (Supplementary Fig. S1E, I, and J).

Altogether, the meta-analysis of public datasets reveals highly inconsistent patterns, with no clear trends across variables (Supplementary Fig. S1E), preventing us to define consensus H4K16ac profiles and to clarify its relationship with gene activity.

### Background-corrected H4K16ac profiling reveals widespread presence across the genome

**Figure 2.**
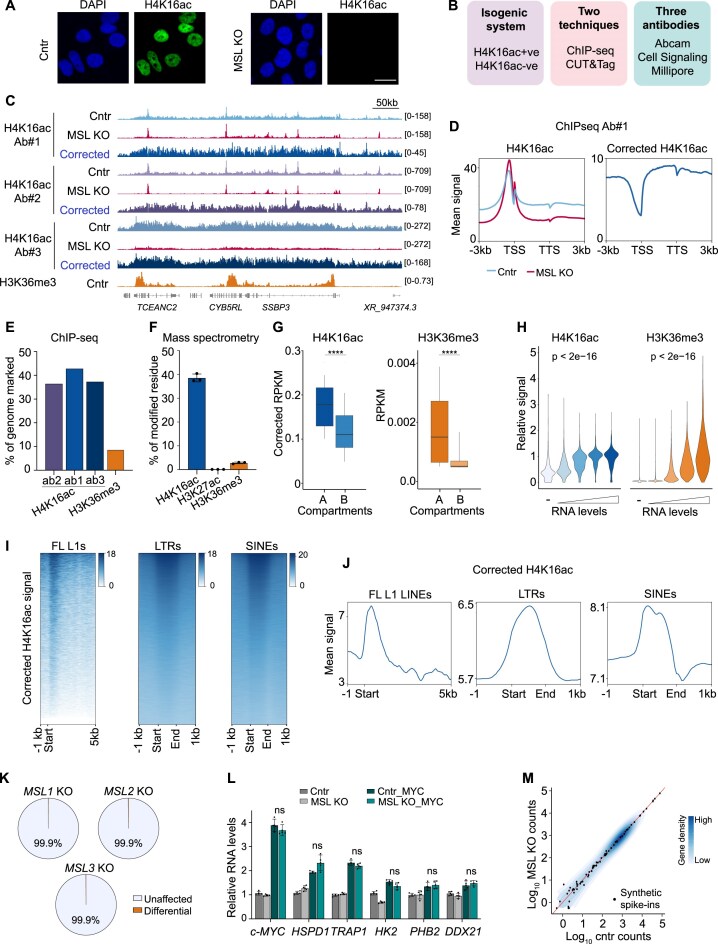
Background-corrected H4K16ac distribution across the genome and impact on transcriptional regulation. (**A**) Immunofluorescence microscopy of HME1 cells showing complete loss of the H4K16ac mark in a *MSL3* KO clone. Cntr: KO of the nonexpressed gene *TNP2*. Scale bars: 20 μm. (**B**) Experimental strategy used in this study to control for possible technical variability affecting H4K16ac profiling. (**C**) ChIP-seq tracks of H4K16ac signal detected in the indicated samples using three different antibodies (Ab) and corresponding background-corrected signal. H3K36me3 is shown as a marker of actively transcribed genes. (**D**) Metaprofiles of H4K16ac ChIP-seq signal (antibody #1) at coding genes, as detected in the indicated cells (left) or after subtraction of KO-derived signal from Cntr signal (right). (**E**) Percentage of the genome (10 kb bins) showing enrichment of the indicated ChIP-seq signal in HME1 cells (see the ‘Materials and methods’ section). (**F**) Percentage of peptides containing the indicated modifications as detected by mass spectrometry analysis of HME1 cells. (**G**) Signal distribution at HiC-defined chromatin compartments of the indicated histone modifications. The corrected signal from antibody #1 is shown for H4K16ac. A, euchromatin; B, heterochromatin. *P*-values from two-sided Wilcox test. (**H**) Distribution of ChIP-seq signal at quartiles of gene expression in HME1 cells. RPKM signal from gene body of corrected H4K16ac ChIP-seq Ab#1 (left) and H3K36me3 ChIP-seq (right) is shown as relative to the mean of the highest gene quartile to enable comparison of the two signals on a common scale. *P*-values from one-way ANOVA. (**I**) Visualization of corrected H4K16ac signal distribution (antibody #1) at repetitive elements. LTR and SINE enriched for the mark are shown. FL L1: full length L1. (**J**) Metaprofiles of H4K16ac signal at different classes of repetitive elements. Corrected signal for antibody #1 is shown. (**K**) Quantification of gene expression changes upon acute inactivation of MSL-complex specific subunits. Genes with |log_2_FC| ≥1 and FDR ≤ 0.01 relative to the control (acute inactivation of the not-expressed gene *TNP2*) were considered differentially expressed. (**L**) Quantification of c-MYC target genes by RT-qPCR data in the indicated samples upon c-MYC overexpression. *N* = 5 biological replicates. *P*-value from Mann–Whitney test. (**M**) Comparison of global mRNA levels in control and MSL KO cells by spike-in-normalized RNA-seq analysis. Every dot is a synthetic ERCC spike-in RNA. Human genes are represented as density plot.

Given the variability observed in public datasets, and to be able to establish causal relationships beyond correlations, we devised a strategy to perturb H4K16ac and examine various nuclear processes in the absence of the mark (Fig. 1A, right). Since MSL disruption in cancer cells promotes genomic instability, which can in turn affect gene expression and other cellular processes confounding the analysis, we opted to use noncancerous hTERT-immortalized human epithelial mammary cells (HME1) as a model system. HME1s have two important features that facilitate our analysis: firstly, unlike cancer cells, they readily tolerate MSL disruption, allowing us to derive monoclonal lines lacking H4K16ac and generate a homogenous isogenic system [7]. Secondly, loss of H4K16ac does not result in CIN, avoiding confounding effects [7]. We derived a H4K16ac-negative clonal population by inactivating the MSL3 subunit of the complex via CRISPR-mediated genome editing (Fig. 2A) (hereafter: MSL KO), and a control population where the nonexpressed gene *TNP2* was targeted by a specific sgRNA.

As a first step to reconcile the conflicting data from previous studies, we profiled H4K16ac in HME1 cells, using the MSL KO line as a control for signal specificity. To account for technical variables, we employed both ChIP-seq and CUT&Tag as profiling methods testing three different antibodies (Fig. 2B and Supplementary Fig. S2A), and included H3K36me3 ChIP-seq as a reference for transcription-associated histone marks. Furthermore, we added *Drosophila* chromatin spike-ins to enable quantitative assessment of global changes (see the ‘Materials and methods’ section).

H4K16ac profiling by ChIP-seq in control cells revealed varying patterns across antibodies. For two of them, we observed a combination of widespread signal and particular enrichment at promoters, while broad enrichment domains dominated when using the third antibody (Fig. 2C and D and Supplementary Fig. S2A). The signal on gene bodies and in intergenic regions was strongly reduced in MSL KO cells, confirming its specificity for H4K16ac; in contrast, the enrichment at gene promoters unexpectedly persisted in H4K16ac-depleted cells, revealing a substantial contribution of nonspecific signal to the profile detected in control cells. After subtraction of the nonspecific signal (see the ‘Materials and methods’ section), we observed a broad H4K16ac distribution without a gene-specific pattern (Fig. 2C and D), consistently across antibodies and profiling methods (Supplementary Fig. S2B). We quantified this widespread occupancy (see the ‘Materials and methods’ section), finding that ∼40% of the genome was marked by H4K16ac, a much greater fraction than observed for H3K36me3 (Fig. 2E). Mass spectrometry analysis confirmed H4K16ac abundance compared to H3K36me3 and other active chromatin marks such as H3K27ac (Fig. 2F).

The corrected H4K16ac signal was consistently enriched in transcriptionally active A compartments regardless of profiling method, but compared to H3K36me3, a substantial signal was also observed in heterochromatic and gene-poor B compartments (Fig. 2G and Supplementary Fig. S2C). At the gene level, we observed a bimodal distribution rather than a linear correlation with mRNA levels (Fig. 2H), suggesting a general association with euchromatin rather than individual gene activity. At a smaller scale, analysis of background-corrected H4K16ac signal confirmed the previously reported enrichment of the mark at TEs [16]. The corrected signal reproducibly showed class-specific profiles, anti-correlating with H3K9me3, as previously shown (Fig. 2I and J, and Supplementary Fig. S2D and E).

We conclude that H4K16ac is broadly distributed across the genome, being present in both euchromatic and heterochromatic domains; at a smaller scale, the mark does not accumulate at genes but is locally enriched at a subset of TEs.

### Loss of H4K16ac does not substantially affect transcriptional regulation

To directly interrogate the contribution of H4K16ac to gene expression regulation in mammalian cells, we leveraged the isogenic nature of the HME1 model and the absence of CIN-induced confounding effects to examine how loss of H4K16ac affects (i) steady-state gene activity; (ii) acutely induced gene expression changes; (iii) global mRNA levels. Inactivation of any MSL subunit impairs complex functionality and results in H4K16ac depletion [7]. Remarkably, regardless of which gene was knocked-out, 99.9% of genes were unaffected (FDR > 0.01 or a log_2_ fold change < |1|) as assessed by RNA-seq analysis (Fig. 2K and Supplementary Table S4), demonstrating H4K16ac dispensability for sustaining steady-state transcription. These results confirm previous observations made in malignant cells, where differential mRNA levels at seemingly misregulated genes were instead the result of DNA copy number changes [7]. We then asked whether H4K16ac loss might impair gene activation upon an acute stimulus. To this end, we overexpressed c-MYC in control and MSL KO HME1 cells and measured the activation of c-MYC targets. c-MYC targets were upregulated in MSL KO cells as efficiently as in control cells, indicating that H4K16ac is not required for acute transcriptional activation either (Fig. 2L). Lastly, given the broad presence of H4K16ac in euchromatin regions, and its established role in chromosome-wide transcriptional activation in *Drosophila*, we examined whether global mRNA levels were affected in H4K16ac-depleted HME1 cells. RNA-seq analysis employing synthetic spike-ins for quantitative normalization did not reveal any global offset in mRNA levels in MSL KO HME1 compared to control cells, ruling out genome-wide effects on mRNA production (Fig. 2M).

Taken together, the combined evidence indicates that the primary function of H4K16ac in human somatic cells is not related to transcriptional regulation.

### H4K16ac loss induces abnormal DNA replication patterns

**Figure 3.**
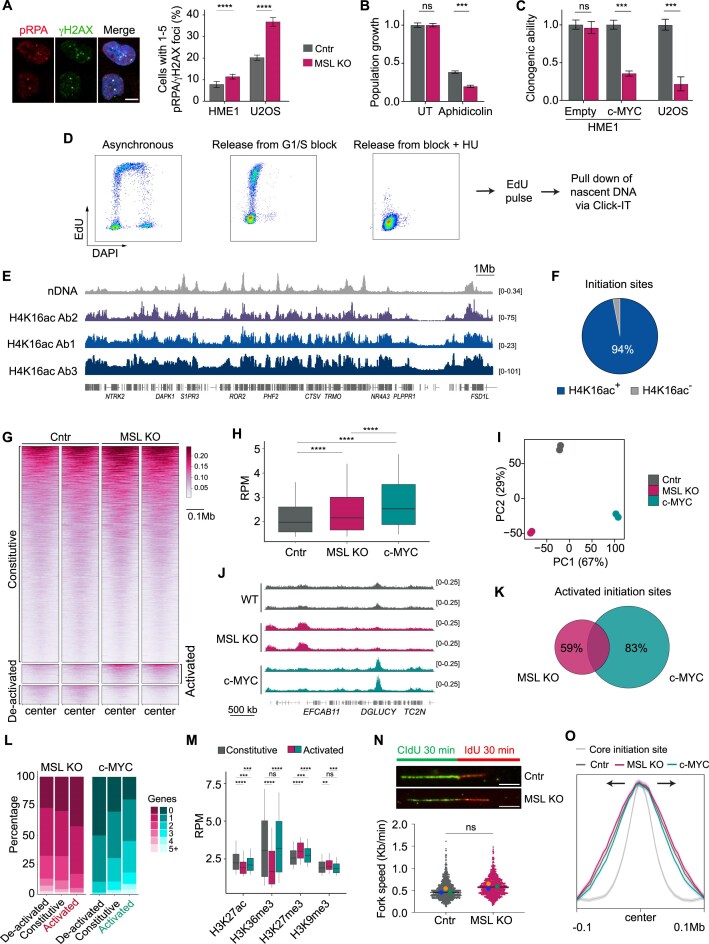
Replication defects in H4K16ac-deficient cells. (**A**) Representative images and quantification of interphase HME1 cells containing double positive pRPA/γH2AX foci in the indicated cell lines, detected by immunofluorescence microscopy. Scale bar: 2 μm. From left to right, *n =* 3162, 3066, 1285, and 718 cells. Values are the mean ± SD from *n* = 3 well areas. *P*-values from two-tailed Fisher’s exact test. (**B**) Aphidicolin sensitivity assay. Values are the mean ± SD from *n*= 3 biologically independent samples after 5 days of growth. *P*-values from unpaired *t*-test with Welch correction. Legend: same as in panel (A). UT: untreated. (**C**) Limiting dilution clonogenic assays quantifying the long-term proliferative capacity of the indicated cell lines. The percentage of populated wells (see the ‘Materials and methods’ section) relative to the corresponding control condition is shown. Values are the mean ± SD from *n* = 3 biologically independent samples. *P*-values from unpaired *t*-test with Welch correction. Legend: same as in panel (A). Empty: cells transduced with the empty control vector. (**D**) Flow cytometry visualizing DNA replicating EdU + cells in the indicated conditions. After synchronization at the G1/S border and release, cells synchronously incorporate EdU into nDNA. In the presence of HU, active origins are arrested soon after firing enabling mapping of replication initiation sites. (**E**) Large-scale distribution of H4K16ac and EdU-HU signals in control HME1 cells. The corrected signal is shown for all H4K16ac antibodies. (**F**) Quantification of replication initiation sites marked by H4K16ac. Data from antibody #1 are shown. Similar percentages were observed with antibody #2. (**G**) Visualization of EdU-HU signal in the indicated cells at replication initiation sites. (**H**). Distribution of EdU-HU signal at the top 50% constitutive replication initiation sites in the indicated cell lines. Mean of the RPM of the 100 kb surrounding the peak summit from two replicates is plotted. *P*-values from two-sided Wilcox test. (**I**) Principal component analysis (PCA) plot showing sample variance between the indicated conditions. The first two components and their percentage contribution are shown. (**J**) Example of differential replication sites showing highly reproducible patterns in biological replicates. (**K**) Overlap of activated replication initiation sites in the indicated conditions. Activated sites are defined as log_2_ fold change ≥ than 0.7, FDR ≤ 10^−10^. (**L**) Relative gene abundance at the indicated groups of replication initiation sites. (**M**) Distribution of the indicated histone modification signals at constitutive and activated (red: MSL KO; green: c-MYC) replication initiation sites. RPM of the 100 kb surrounding the replication peak summit are plotted. *P*-values from two-sided Wilcox test. See the ‘Materials and methods’ section for ChIP-seq data sources. (**N**) DNA fiber assay. Top: Representative DNA fiber images showing CldU (green) and IdU (red) incorporation in Cntr and MSL KO cells. Bar = 5 μm. Bottom: Quantification of fork speed (kb/min). Overlaid dots: Averages from three independent experiments. Horizontal bars: Median of all data points. Ns, nonsignificant (unpaired Student’s *t*-tests assessing the difference in means of two data groups). (**O**) Metaprofiles of EdU signal showing unaffected replication fork progression in MSL KO cells. Merged replicate tracks are visualized as mean ± standard error of the mean.

Having ruled out a transcriptional role for H4K16ac, and considering previous findings that its loss enhances genomic instability in cancer cells [7], we examined a possible function for the mark in genome maintenance. Even though H4K16ac-depleted HME1s do not exhibit CIN-associated phenotypes, such as accumulation of micronuclei or anaphase bridges [7], we could detect signs of mild replication stress: the MSL KO population showed a 1.2-fold increase in the fraction of cells containing pRPA/γH2A.X foci (Fig. 3A), indicative of replication defects [51]. The increase was small, and did not even reach the basal levels detected in MSL-proficient U2OS cancer cells, but was significant (*P* <.0001, two-tailed Fisher’s test) and detected reproducibly (Fig. 3A). In line with the notion that loss of H4K16ac does not promote CIN in noncancerous, genomically stable cells, MSL KO HME1s displayed unaffected fitness under unperturbed conditions (Fig. 3B and Supplementary Fig. S4A). However, when we challenged cells to induce additional replication stress, either by treatment with the DNA polymerase inhibitor Aphidicolin (Fig. 3B) or by expressing the *c-MYC* oncogene (Fig. 3C), H4K16ac-depleted HME1s exhibited compromised fitness, mirroring the synthetic lethal phenotype observed in genomically unstable cancer cells (Fig. 3C) [7]. These observations provide initial evidence that H4K16ac may be involved in the DNA replication process. The mild replication stress observed in the absence of H4K16ac is inconsequential in normal unchallenged cells, but when replication stress is exacerbated, deleterious phenotypes are exposed.

To further investigate the relationship between H4K16ac and DNA replication, we employed EdU-HU-seq to map nDNA in the presence or absence of the mark. After synchronization of cells at the G1/S border by treatment with a CDK4/6 inhibitor, we released them into S phase in the presence of HU to prevent replication fork progression, and labeled replication initiation sites with a short EdU pulse (Fig. 3D). In agreement with data obtained in other experimental systems using a similar approach [48], we identified 2585 replication initiation zones with an average size of 250 kb (Supplementary Fig. S3A). Comparison of large-scale nDNA and H4K16ac profiles revealed a striking similarity, with 94% of replication initiation zones being marked by H4K16ac (Fig. 3E and F). We then compared replication initiation patterns in control and MSL KO cells. As a positive control, we included in the analysis c-MYC-overexpressing cells, since the oncogene induces replication stress via hyperactivation of replication origins and consequent fork collapse [48]. For each condition, we defined constitutive and differential zones, either activated or deactivated upon H4K16ac loss or c-MYC overexpression (Fig. 3G, Supplementary Fig. S3B, and Supplementary Table S5). We observed two aberrant phenotypes in H4K16ac-depleted cells: first, the strongest constitutive replication zones (top half peaks by magnitude) were significantly hyperactivated (*P* <.0001) (Fig. 3H). The effect size was smaller compared to c-MYC-overexpressing cells, but the trend was comparable (Fig. 3H and Supplementary Fig. S3B). Second, 175 sites were exclusively detected in MSL KO cells, corresponding to *de novo* initiation zones that were activated in the absence of H4K16ac, reaching nDNA levels comparable to constitutive sites (Fig. 3G and J). A small subset of deactivated zones was also detected, but since their basal activity in control cells was low, we reasoned that their deactivation would likely have minimal impact (Fig. 3G). c-MYC overexpressing cells showed similar patterns (Supplementary Fig. S3B), but the overall profile was distinct from that of H4K16ac-depleted cells, as shown by PCA (Fig. 3I). Differential H4K16ac-related sites were largely nonoverlapping with c-MYC-related ones (Fig. 3J and K, and Supplementary Fig. S3C) and were located in distinct genomic domains: while c-MYC-activated sites, as expected, were in gene-rich and euchromatic regions enriched for active histone marks [52], H4K16ac loss induced aberrant replication initiation in gene-poor regions enriched for repressive marks (Fig. 3L and M). Activation of *de novo* replication initiation zones in the absence of H4K16ac was not a compensatory response to slow fork progression across the genome, as revealed by DNA fiber assays (Fig. 3N and Supplementary Fig. S4B). In agreement, the EdU incorporation rate from core initiation zones after HU release was indistinguishable in control and MSL KO cells (Fig. 3O and Supplementary Fig. S4C) (see the ‘Materials and methods’ section).

Thus, H4K16ac loss induces replication stress by hyperactivating constitutive replication initiation zones and by activating *de novo* sites in heterochromatic, gene-poor domains.

Since other histone modifications are largely unaffected in MSL-KO cells, as shown by quantitative histone mass spectrometry (Supplementary Fig. S4D and E) [5], these effects appear to be mediated either directly by the loss of H4K16ac, or possibly by a related increase in the nearby H4K12ac, a mark known to promote origin MCM loading [53–55].

### H4K16ac inhibits the activation of cryptic DNA replication origins at LTRs

Given the local enrichment of H4K16ac at specific TEs, we wondered whether TEs could be involved in the altered replication patterns seen upon H4K16ac loss.

**Figure 4.**
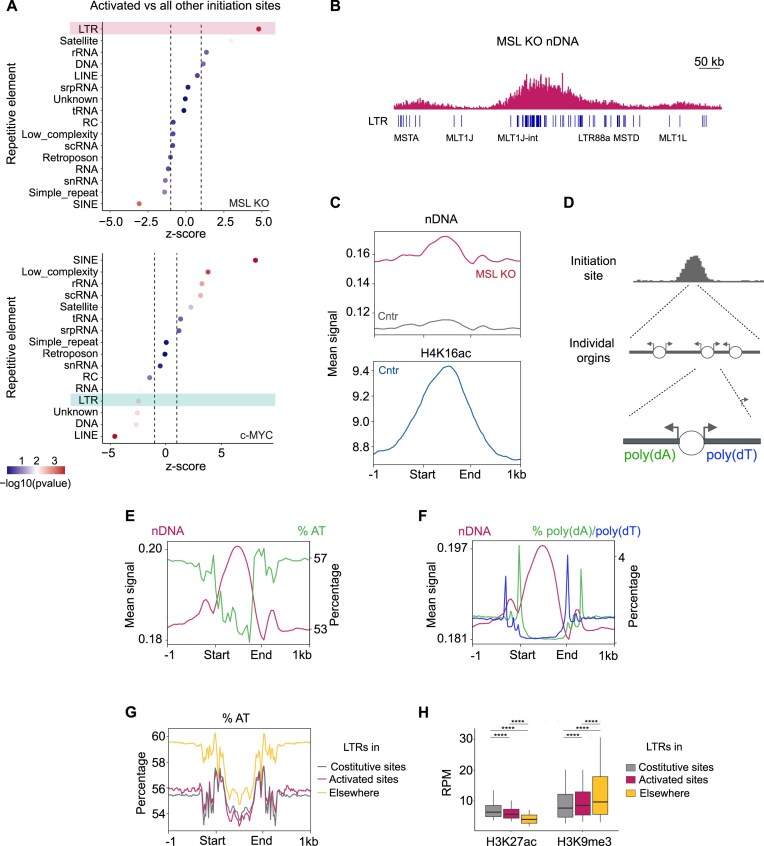
LTRs are cryptic replication origins repressed by H4K16ac. (**A**) Enrichment analysis of repetitive elements at replication initiation sites activated upon MSL KO (top) or c-MYC overexpression (bottom). Enrichment is calculated through a permutation test comparing activated initiation sites to all the other replication initiation sites. (**B**) Track showing LTR enrichment at a replication initiation site activated in MSL-KO cells. (**C**) Metaprofiles of EdU and H4K16ac (corrected ChIP-seq with antibody #1) signals in the indicated conditions at LTR overlapping replication initiation sites. Signals are stranded based on LTR orientation. (**D**) Schematic illustrating the relationship between initiation sites and individual replication origins at different scales, and the nucleotide compositions of origin-flanking regions that favors their activation. Based on data from Tubbs *et al.* [44]. (**E, F**). Metaprofiles of the indicated features at LTRs. The profiles of AT content (E) and poly(dA)/poly(dT) tracts (F) are shown relative to the EdU-HU signal detected in H4K16ac-depleted cells. Signals are stranded based on LTR orientation. (**G**) AT content profiles at the indicated LTR subsets. (**H**) Distribution of the indicated histone modification signals at LTR subsets. *P*-values from two-sided Wilcox test. See the ‘Materials and methods’ section for ChIP-seq data sources.

By comparing the relative abundance of different TE classes in activated vs constitutive replication initiation zones, we found a strong enrichment of LTR elements in sites activated in H4K16ac-deficient cells (Fig. 4A and B), contrasting with their depletion in c-MYC-activated zones (Fig. 4A, bottom). This pattern was specific to LTRs, with LINEs showing no enrichment and SINEs being significantly depleted from *de novo* sites (Fig. 4A). LTRs are retrotransposons belonging to the Class I of TEs, which also contains the non-LTR retrotransposons LINEs and SINEs [56]. LTRs range in size from ∼100 to ∼1000 bp and make up ∼8% of the genome. Given the striking enrichment of LTRs in activated replication initiation zones, we examined nDNA profiles at high resolution within those sites. In control cells, the nDNA signal at LTRs was close to background levels, resulting in a flat profile. In contrast, MSL KO displayed a local accumulation of nDNA at LTRs (Fig. 4C, top), with a profile that mirrored the lost H4K16ac enrichment (Fig. 4C, bottom; Supplementary Fig. S5A). Notably, both nDNA and H4K16ac profiles at LTRs were asymmetric—particularly visible for the mark in the CUT&Tag dataset owing to its higher resolution (Supplementary Fig. S5A). This observation raises the possibility that, in the absence of H4K16ac, individual LTRs may be used as DNA replication origins, with clusters located in specific genomic regions driving activation of *de novo* initiation zones. Further supporting a role for H4K16ac in repressing LTR usage as replication origins, cells lacking SIRT1, the main H4K16ac deacetylase, showed opposite patterns compared to MSL KO cells, displaying decreased nDNA levels at LTRs (Supplementary Fig. S5B). Activation of cryptic origins in H4K16ac-deficient cells was not accompanied by changes in repressive histone marks (Supplementary Fig. S5C).

Replication initiation zones contain multiple DNA replication origins, small regions of a few hundred base pairs where the replicative helicase (MCM2-7) assembles and unwinds the parental DNA duplex to establish bidirectional replication [57] (Fig. 4D). In mammals, replication origins are degenerate and fire stochastically within initiation zones across individual zones [58, 59]. Compared to bacteria or lower eukaryotes, mammalian origins lack a strict consensus sequence, but they are often characterized by a distinct GC content and nucleosome depletion [60]. In particular, firing origins tend to be flanked by antiparallel dA/dT tracts, which facilitate DNA unwinding by destabilizing DNA–nucleosome interactions [44]. Examining LTRs, we found a striking resemblance with sequence patterns observed at replication origins: LTR bodies across the genome were depleted of AT nucleotides, but flanked by AT-rich regions at both ends, with a profile remarkably symmetrical to the nDNA one detected by EdU-HU-seq in MSL KO cells (Fig. 4E). Furthermore, we observed sharp peaks of asymmetric poly(dA)/poly(dT) tracts (20 nucleotide stretches with a minimum content of A or T of 75%) at either side of the nDNA-enriched LTR body, mirroring the sequence context that defines canonical replication origins (Fig. 4F).

These observations suggest that LTRs can act as cryptic DNA replication origins, and that some of them are used in the absence of H4K16ac. We then investigated what determines which LTRs are activated. We found that LTRs located in *de novo* replication zones are characterized by a lower surrounding AT content and more pronounced flanking enrichment in the immediate vicinity of the body (Fig. 4G), similar to those present in constitutive origins. In contrast, LTRs located elsewhere in the genome (i.e. not used) showed a distinct pattern, with an overall higher AT content, both in the surrounding regions and in the LTR body (Fig. 4G). Furthermore, activated LTR-associated origins were located in genomic regions with a distinct epigenetic state, characterized by intermediate H3K27ac and H3K9me3 levels compared to constitutive origins and inactive LTRs (Fig. 4H).

Altogether, these observations indicate that LTRs, because of their sequence composition or integration preference across the genome, have the potential to initiate DNA replication, but they are typically repressed by H4K16ac, either directly or indirectly. In the absence of H4K16ac, a subset of them that resemble canonical origins at the genetic and epigenetic level get activated.

### Synergistic effect of oncogenes and H4K16ac loss at LTR-associated cryptic origins

Our previous findings indicate that pre-existing replication stress in cancer cells synergizes with H4K16ac loss, promoting CIN [7]. We therefore examined whether oncogene-induced replication stress promotes usage of LTRs as replication origins, independently of H4K16ac. Providing initial support, HCT116 colon cancer cells and U2OS sarcoma cells profiled by EdU-HU-seq [48, 61] showed enrichment of nDNA at LTRs despite the presence of H4K16ac (Supplementary Fig. S5D). Accumulation of nDNA at LTRs was also detected in cancer cells profiled using alternative origin mapping methods [Nascent strand DNA sequencing (NS-seq), Okazaki fragment sequencing (OK-seq), Short nascent strand sequencing (SNS-seq) and Initiation site sequencing (Ini-seq2)] [20, 47, 62] (Supplementary Fig. S5E). To more directly assess the effect of oncogene-induced replication stress, we leveraged our ability to temporally control c-MYC expression in noncancerous HME1 cells, in the presence or absence of H4K16ac. LTR-associated cryptic origins that responded to H4K16ac loss also responded to oncogene activation, displaying nDNA accumulation (Supplementary Fig. S5F). Furthermore, simultaneous expression of the oncogene and loss of the mark further increased nDNA levels, maximizing the aberrant use of LTRs as replication origins (Supplementary Fig. S5F). Together with the observation that MSL disruption is tolerated by normal HME1 cells but decreases the fitness of oncogene-expressing cells (Fig. 3C) [7], this data suggests that activation of LTR-associated cryptic origins becomes deleterious only beyond a certain threshold.

### Disrupted temporal regulation of genome duplication in the absence of H4K16ac

**Figure 5.**
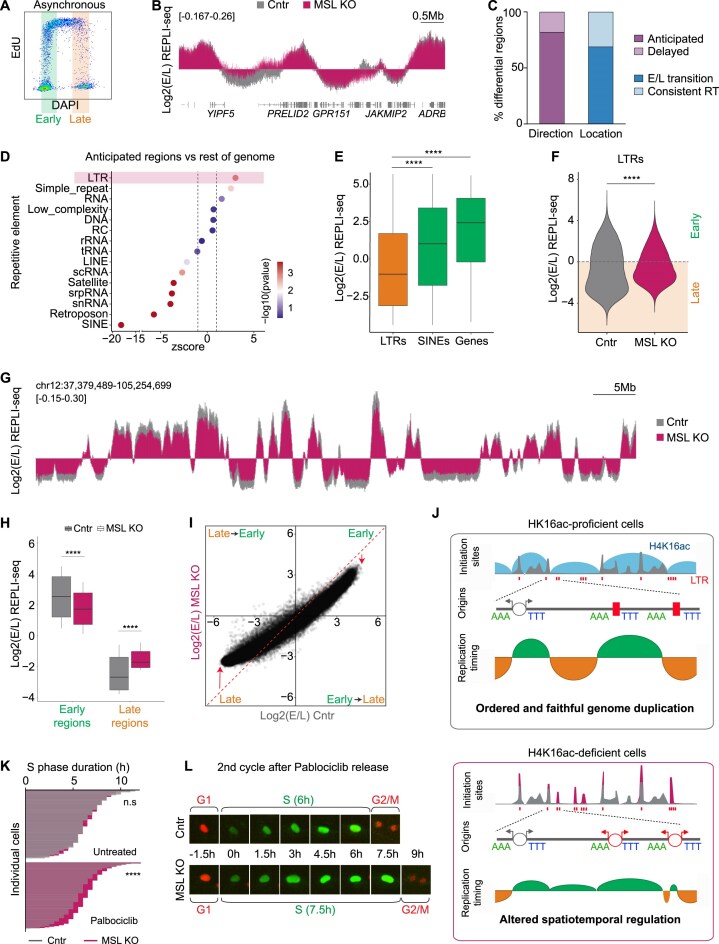
Altered RT in H4K16ac-deficient cells. (**A**) Flow cytometry of asynchronous cells illustrating the subset of cells analyzed by REPLI-seq upon cell sorting. (**B**) Overlay of REPLI-seq tracks from control and H4K16ac-deficient HME1 cells (see also Supplementary Fig. S6A). (**C**) Quantification of the indicated features in regions displaying differential RT in H4K16ac-deficient HME1 cells (see the ‘Materials and methods’ section for definitions). (**D**) Enrichment analysis of repetitive elements at anticipated regions upon H4K16ac loss. Enrichment is calculated through a permutation test comparing anticipated regions to random regions in the genome. (**E**) Distribution of RT index of 50 kb genomic bins overlapping the indicated element, as measured in control HME1 cells. *P*-values from two-sided Wilcox test. (**F**) Distribution of RT index of LTRs in the indicated cell lines. *P*-values from two-sided paired Wilcox test. (**G**) Track showing the RT of chromosome 12’s long arm in the indicated cell lines, highlighting the global effect of H4K16ac loss. (**H**) Distribution of RT index at 50 kb bins across the genome in the indicated cell lines. Bins are classified as early or late based on the RT index measured in control cells. *P*-values from two-sided paired Wilcox test. (**I**) Genome-wide relationship between RT indexes measured in control and H4K16sc-depleted cells. Each dot is a 50 kb genomic bin. Note the overall tilted pattern relative to the diagonal, and the greater differences observed in late vs early regions (red arrows). (**J**) Model of how H4K16ac preserves genome stability by repressing LTR-associated AT-flanked cryptic replication origins. In its absence, premature replication of LTR enriched heterochromatic regions alters the temporal control of genome duplication. Created in BioRender. Milan, M. (2025), https://BioRender.com/n87p237. (**K**) S phase duration in individual cells of the indicated FUCCI(CA)2-expressing cell lines. *P*-values from one-way ANOVA. (**L**) Time-lapse images of representative FUCCI(CA)2-expressing cells showing prolonged S phase in H4K16ac-deficient cells in the second cycle after Palbociclib release.

Because of the widespread presence of LTRs across the genome, we reasoned that their aberrant use as replication origins may have a broad impact on the replication program. To investigate, we used REPLI-seq [63] to map genome-wide RT during S phase, in the presence or absence of H4K16ac. After sorting cells in early or late S phase (Fig. 5A) and sequencing EdU-labeled nDNA, we measured RT as the log_2_ ratio of early-to-late reads, and overlaid profiles detected in control and MSL KO cells for direct comparison (Supplementary Fig. S6A).

We identified 2292 differential regions (*P* <.01) (see the ‘Materials and methods’ section), most of which (82%) displayed earlier RT in H4K16ac-depleted cells (Fig. 5B and C). These regions included sites that flipped from late to early RT, and others that maintained late RT but were replicated earlier in the absence of H4K16ac (Fig. 5B and Supplementary Fig. S6A). Interestingly, about 69% of the differential regions were located at the transition between early- and late-replicating domains, suggesting a less tight compartmentalization of replicating regions in H4K16ac-depleted cells (Fig 5C and Supplementary Fig S6B, boxed area). Notably, regions with anticipated RT were significantly enriched for LTRs (*P* <.005) (Fig. 5D). Across the genome, we found LTRs were mostly located in late-replicating domains, as defined in control cells, in contrast to SINEs which were enriched in gene-rich, early-replicating ones (Fig. 5E). Beyond statistically defined differential regions, LTRs RT was broadly affected in MSL KO cells, with LTRs located in late-replicating domains in control cells showing earlier replication in the absence of H4K16ac (Fig. 5F), consistent with the EdU-HU-seq data. As a control, the RT distribution at SINEs was only minimally affected (Supplementary Fig. S6C).

The human genome contains about half a million LTRs and premature replication at many of these elements in H4K16ac-depleted cells resulted in global RT alterations. At chromosome-scale, MSL KO cells exhibited flattened RT profiles, with both positive and negative domains closer to the middle line (Fig. 5G). Since the magnitude of replicating domains manifests the highly coordinated replication program during the S phase across individual cells [64], the altered pattern observed in H4K16ac-depleted cells indicates a reduced temporal control of the DNA replication program. Large-scale RT alterations impacted both early- and late-replicating domains across all chromosomes (Fig. 5G–I and Supplementary Fig. S6D), but changes were more substantial in late-replicating regions, which are enriched for LTR (Fig. 5I, red arrows). Together with the identification of *de novo* initiation zones in LTR-enriched heterochromatic regions, this observation suggests that loss of H4K16ac primarily affects late-replicating domains by promoting their premature replication, and that a secondary “domino” effect interferes with early-replicating domains, likely due to competition for limiting replication factors [65, 66].

Collectively, these findings suggest a model whereby H4K16ac ensures an ordered genome replication program by preventing activation of LTR-associated, AT-flanked replication origins (Fig. 5J). In its absence, premature replication of LTR-enriched heterochromatic regions, in turn, delays the replication of euchromatic domains (Fig. 5J). Remarkably, these defects are inconsequential in normal cells (Fig. 3B and C, and Supplementary Fig. S4A), and only when RT alterations synergize with oncogene-induced replication stress in cancer cells, they are exposed and affect cell fitness (Fig. 3B and C), as previously observed [7]. This model predicts that normal H4K16ac-depleted HME1s should exhibit unaffected S phase duration, but that exacerbation of RT defects should prolong the S phase, as cumulative disorder in executing the program hinders efficient genome replication. To test the model, we employed the FUCCI (fluorescent ubiquitination-based cell cycle indicator) reporter [67] to monitor S phase kinetics at the single-cell level (Supplementary Fig. S6E). In line with the lack of fork progression defects in the absence of H4K16ac (Fig. 3N and O, and Supplementary Fig. S4B and C), untreated MSL KO HME1 cells progressed through S-phase with comparable kinetics as control cells (S-phase duration: 6 ± 2 h) (Fig 5K, top). In contrast, upon treatment with palbociclib, a CDK4/6 inhibitor known to alter RT once cells resume cycling [68], individual MSL KO cells consistently exhibited a delay in S-phase exit (45 min ± 34) (Fig. 5K bottom and 5L), suggesting that additive RT defects resulted in overt replication stress and delayed completion of genome duplication. Altogether, these findings uncover a critical role for H4K16ac in maintaining genome integrity by ensuring spatiotemporal fidelity of the replication program.

## Discussion

Histone acetylation across various residues has widely been implicated in transcriptional activation. Based on genome-wide enrichment of most acetylated marks at active genes, the broad transcriptional effects of HDACi, and *in vitro* observations that lysine acetylation destabilizes nucleosome DNA interactions [3, 12], it has been proposed that histone acetylation mainly promotes local chromatin decondensation to facilitate the recruitment and activity of transcription factors at target genes. H4K16ac is one of the most abundant histone acetylation marks, present on nearly half of the nucleosomes across the genome (Fig. 2E and F) [5]. While its role in dosage compensation in *Drosophila* has been extensively characterized, its function in mammalian cells has remained elusive. In this study, we demonstrate that, in human somatic cells, H4K16ac contribution to transcriptional regulation is minimal—if any at all. Several lines of evidence support this conclusion: (i) background-corrected H4K16ac signal is not enriched at genes; (ii) across many datasets, gene activity does not correlate with H4K16ac levels; (iii) loss of H4K16ac does not substantially affect gene expression profiles, global mRNA levels, or the ability to upregulate genes in response to stimuli. In agreement, H4K16ac-depleted cells show unaltered chromatin accessibility profiles [5]. While particularly surprising for H4K16ac, given its established role in X-chromosome hyperactivation in flies [10], the observed uncoupling from gene expression regulation aligns with findings on other histone marks, such as H3K27ac, which are dispensable for transcriptional activation despite broad enrichments at active regulatory elements [69–71]. Importantly, previous studies linking H4K16ac loss to transcriptional defects in MOF/KAT8-deficient cells [72] need to be interpreted with caution: KAT8 loss also disrupts the NSL complex, a regulator of housekeeping genes, whose disruption also alters H4K8ac and H4K5ac and has broadly deleterious effects [5]. Our data do not rule out a transcription-related function for H4K16ac during embryonic development.

We demonstrate that H4K16ac regulates the temporal order of genome duplication. The link between histone acetylation and DNA replication was first established in budding yeast, where dynamic acetylation and deacetylation of H3 and H4 at regions flanking DNA replication origins were shown to be essential for efficient origin activation [73]. Specifically, H4K16ac has been reported to be deposited at active origins concomitant with, or potentially immediately preceding, the appearance of nDNA [18]. Consistent with these findings, we observed that virtually all DNA replication initiation zones in human cells are enriched for H4K16ac. This enrichment might suggest a model in which H4K16ac-induced chromatin decompaction facilitates DNA replication by promoting the binding of replication machinery or by aiding fork progression. However, two key observations challenge this model: firstly, H4K16ac-depleted cells show an increase, rather than a decrease, in nDNA levels. Secondly, these cells exhibit no detectable changes in fork progression rates, in line with unaffected S phase kinetics and cell proliferation rate. Instead, we show that the loss of H4K16ac disrupts the spatiotemporal regulation of genome duplication by enabling aberrant activation of subsets of LTRs as replication origins and promoting premature replication of heterochromatic regions across the genome. The precise mechanism by which H4K16ac prevents activation of AT-flanked, LTR-associated origins remains unclear. Given its ability to modulate internucleosomal interactions and inhibit the chromatin remodeler ISWI [12], one possibility is that H4K16ac might indirectly contribute to restricting accessibility of replication factors to LTR-enriched heterochromatic regions by broadly influencing nucleosome positioning. An alternative scenario is that cross-talk between H4K16ac and other histone marks may underscore the observed phenotypes: H4K20me2 is a critical modification that recruits the Origin Recognition Complex (ORC) on chromatin to mediate origin licensing [74]. It is plausible that adjacent H4K16ac may interfere with ORC binding at LTRs, thereby inhibiting their licensing as replication origins. Alternatively, as suggested by our Mass Spectrometry (MS) analysis, H4K16ac may prevent accumulation of H4K12ac at LTRs, thereby hindering MCM loading [53–55]. Future studies will be necessary to elucidate the molecular mechanisms underlying the inhibition of LTR-associated origins by H4K16ac and to determine how this modification integrates with other epigenetic mechanisms to maintain the fidelity of the DNA replication program. Our data cannot formally rule out that unknown substrates of MSL may underpin the replication defects observed in MSL KO cells, and approaches such as histone editing [71] would be required to definitively prove the causal role of H4K16ac. However, the opposite patterns observed in MSL KO and SIRT1 KO cells, together with the local and global correlations between H4K16ac and nDNA profiles, provide strong correlative evidence that the observed phenotypes are likely mediated by loss of the mark.

Despite broad alterations in RT induced by H4K16ac loss, normal human cells progress through S phase with unaffected kinetics and maintain fitness comparable to H4K16ac-proficient cells. This phenotype mirrors observations in cells lacking RIF1, a master regulator of the RT program in mammalian cells, which primarily represses replication origins in late-replicating heterochromatic domains [64]: in RIF1-knockout cells, RT profiles are flattened due to high heterogeneity in origin activation across individual cells, yet the population as a whole proceeds through the cell cycle with minimal disruptions [64]. These findings suggest that tight temporal control of origin activation is not essential for genome duplication in unchallenged cells, and that the replication program is intrinsically flexible at the single-cell level. However, under conditions of replication stress—such as following oncogene activation—this flexibility becomes insufficient. Replication factors likely become limiting, and the resulting defective genome duplication leads to DNA damage and CIN [7].

In conclusion, our findings reveal an unexpected function for one of the most abundant chromatin modifications, and uncover a novel regulatory mechanism that ensures faithful genome replication. By providing a mechanistic explanation for the synthetic lethal phenotype observed in cancer cells upon MSL disruption, our results also suggest potential strategies to exacerbate replication stress beyond tolerable thresholds, offering new opportunities to specifically target malignant phenotypes.

## Supplementary Material

gkaf916_Supplemental_Files

## Data Availability

Sequencing data generated in this study have been deposited at NCBI-GEO (Super-Series GSE286138). Public datasets are listed in Supplementary Table S3. The mass spectrometry data have been deposited to the ProteomeXchange Consortium via the PRIDE partner repository with the dataset identifiers PXD039819 (public) and PXD066155.
