# Supplementary material for: Mammalian H4K16ac regulates the spatiotemporal order of genome replication rather than gene expression
Source: Nucleic Acids Res. 2025 Sep 23;53(18):gkaf916. doi: 10.1093/nar/gkaf916 (PMC12455591; doi:10.1093/nar/gkaf916)
Supplement: gkaf916_Supplemental_Files [file gkaf916_supplemental_files.zip › Suppl._Figures_+_legends_Rev.pdf]

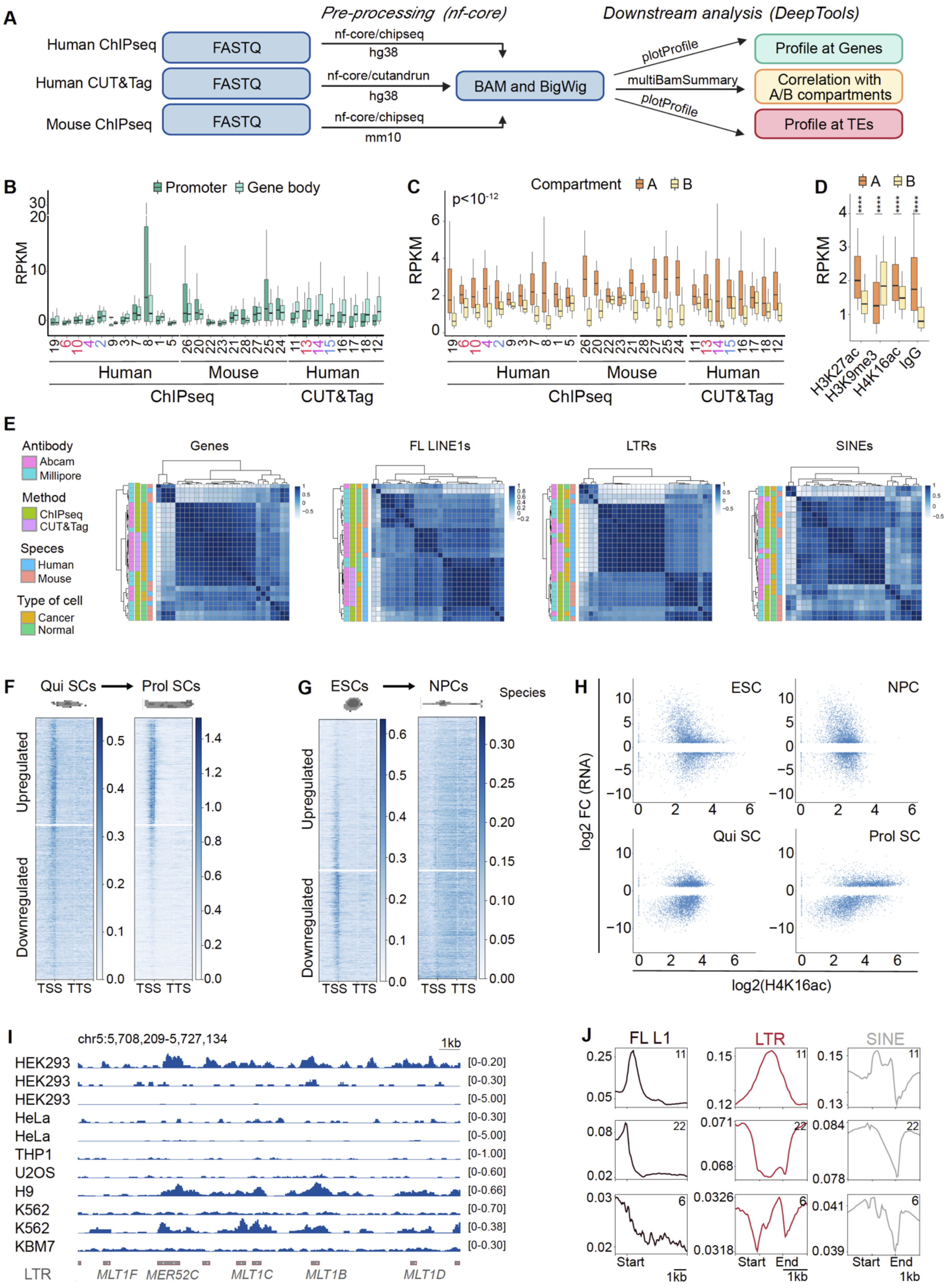

Figure S1: Standardized analysis of public H4K16ac datasets

A. Schematic of the standardized analysis carried out on public datasets. Created in BioRender. Milan, M. (2025) https://BioRender.com/s1itb0o **B**. Comparison of H4K16ac signal intensity at promoters (1 kb upstream of TSS) and gene bodies across the analyzed datasets. RPKM, reads per kilobase per million. X-axis: dataset number matches figure 1B. Cell lines probed in multiple studies are color-coded. **C**. H4K16ac signal distribution

at HiC compartment in the analyzed datasets. A, euchromatin; B, heterochromatin; RPKM, reads per kilobase per million. P-values from two-sided Wilcox test on the medians. X-axis and color-coding: same as (B). **D**. Signal distribution at HiC compartment of the indicated histone modifications in H9 cells (human embryonic stem cells) from dataset GSE200770. H4K16ac: same as sample 11 in panel C. P-values from two-sided Wilcox test, corrected for multiple testing. **E**. Spearman correlation of H4K16ac signal distribution at the indicated elements. All datasets indicated in Figure 1B are present. **F**. Visualization of H4K16ac signal at genes differentially expressed in the transition from quiescent (Qui SC) to proliferating satellite cells (Prol SC), sorted by fold change. N= 4940. **G**. Visualization of H4K16ac signal at genes differentially expressed in the transition from embryonic stem cells (ESC) and neural progenitor cells (NPC), sorted by fold change in gene expression. N= 4757. **H**. Relationship between H4K16ac signal (RPKM at promoter -1kb upstream of TSS- and gene body) and fold change of differentially expressed genes. Each dot is a gene. N and abbreviations as in (F) and (G). **I**. H4K16ac tracks showing high variability in enrichment at LTR among datasets. **J**. Metaprofiles of H4K16ac signal at different classes of repetitive elements. Each line represents a different dataset. Dataset number in the upper right corner: same as Fig. 1B. FL L1: full length L1.

A

	Antibody #1	Antibody #2	Antibody #3
Provider	Millipore	Cell Signaling	Abcam
Cat. number	07-329	13534	ab109463
Profiling method	ChIP-seq	ChIP-seq	ChIP-seq CUT&Tag

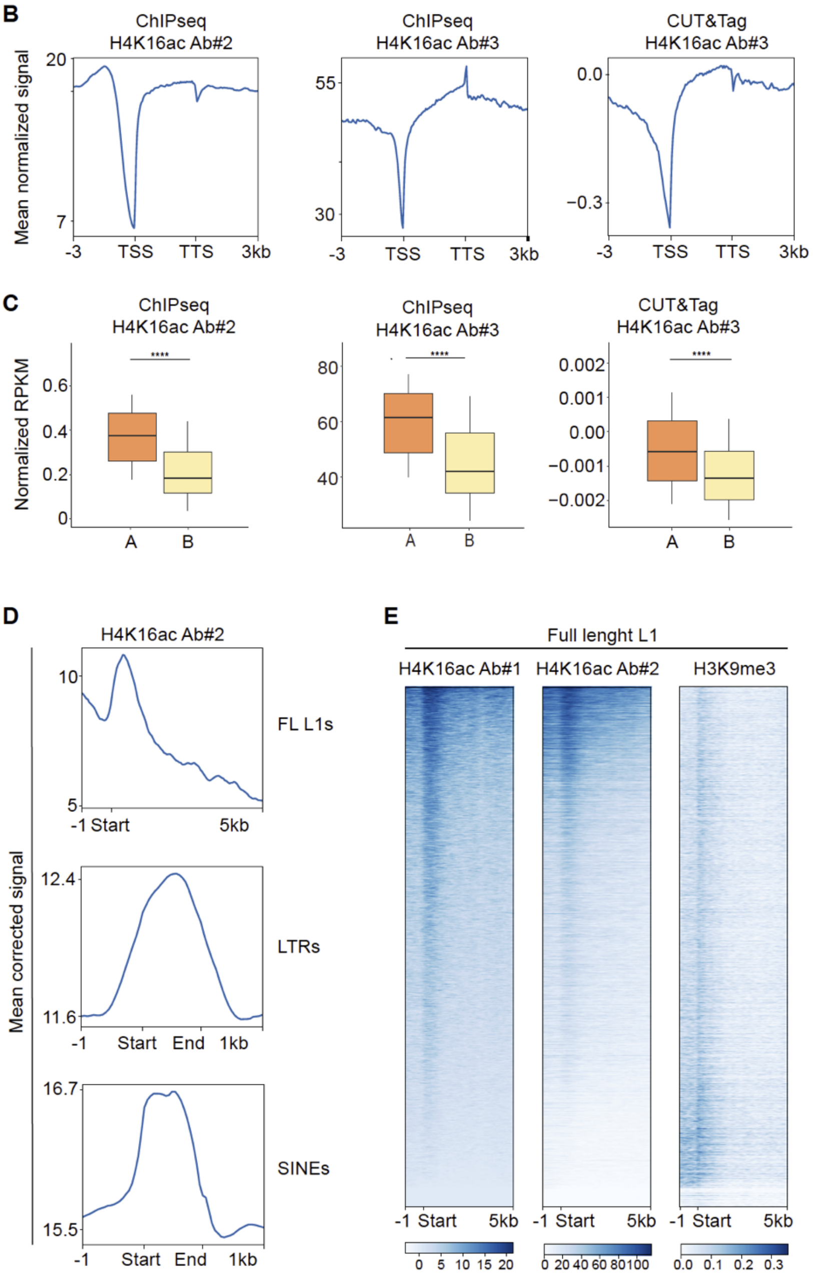

Figure S2: Reproducibility of background-corrected H4K16ac profiles across antibodies and profiling methods

A. Table describing the H4K16ac antibodies used in this study. **B**. Metaprofile of the indicated H4K16ac signal at coding genes. Corrected signal over KO-derived signal is plotted. TSS, transcription start site; TTS, transcription termination site. **C**. H4K16ac signal distribution at HiC-defined chromatin compartments as profiled with the indicated methods and antibodies. A, euchromatin; B, heterochromatin; RPKM: reads per kilobase per million. P-values from two-sided Wilcox test. **D**. Metaprofiles of H4K16ac ChIP-seq signal at the indicated classes of repetitive elements. FL L1: full length L1. Corrected signal for antibody#2 is plotted. **E**. Relationship between the indicated histone marks at full length L1 as detected by ChIP-seq. Elements are sorted by signal intensity in the H4K16ac dataset obtained with antibody#1. Corrected H4K16ac is shown for both H4K16ac antibodies. H3K9me3 ChIP-seq data from GSE29611.

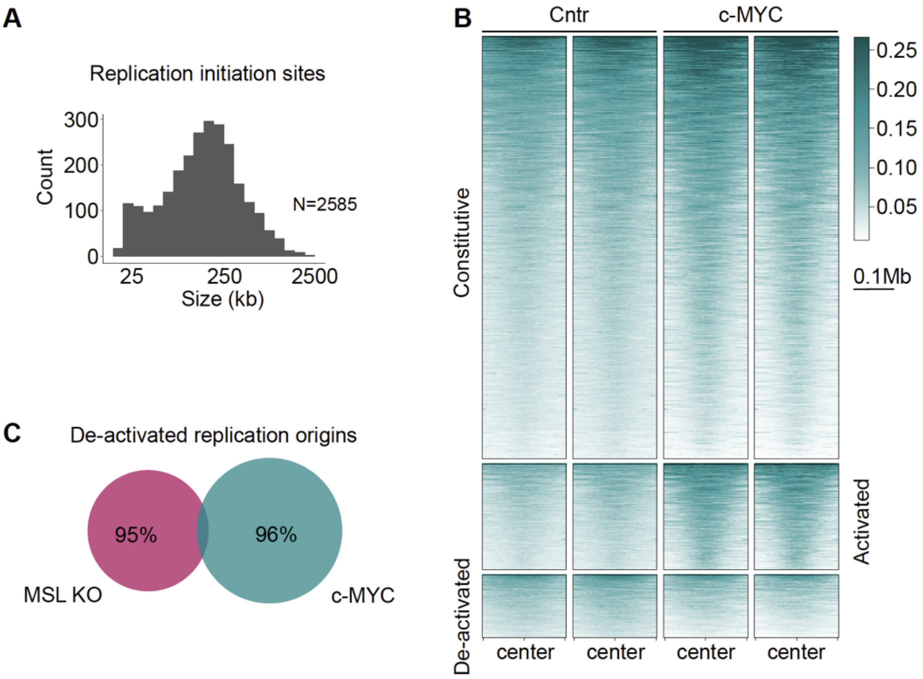

Figure S3: Nascent DNA profiles in c-MYC overexpressing HME1s.
A. Size distribution of replication initiation sites in HME1 cells as detected by EdU-HU-seq. **B**. Visualization of EdU-HU signal in control and c-MYC-overexpressing cells at replication initiation sites. **C**. Overlap of de-activated replication initiation sites in the indicated conditions. De-activated sites are defined as $\log_2$ fold change ≤ -0.7, FDR ≤ 10^{10}.

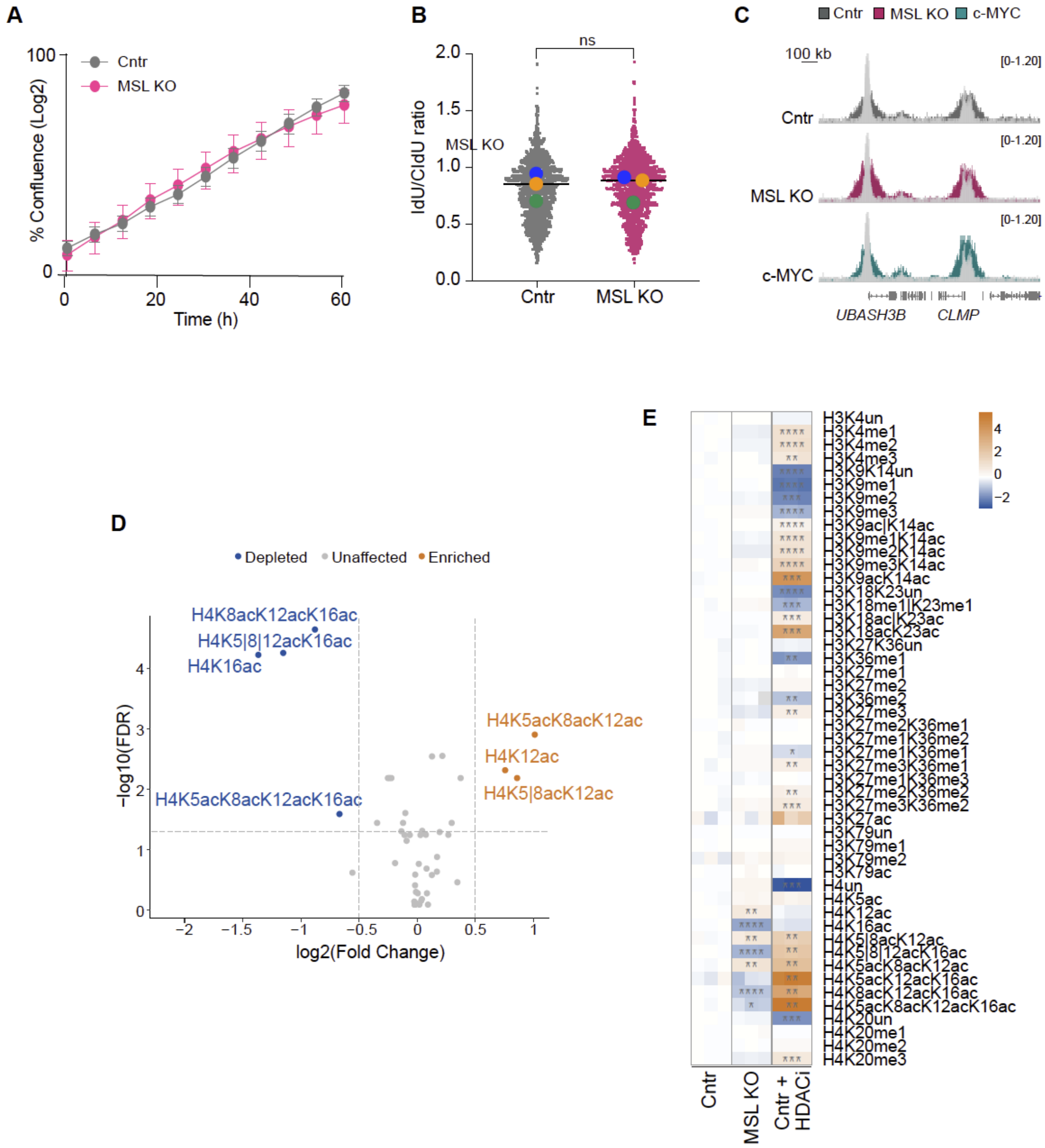

Figure S4: H4K16ac impact on fork progression and chromatin modifications

A. Cell proliferation curves of the indicated HME1 lines. **B**. DNA fiber assay in Cntr and MSL KO HME1 cells. Overlaid dots: averages from three independent experiments. Horizontal bars: median of all data points. Ns: non-significant (Unpaired t-tests assessing the difference in means of two data groups). **C**. Tracks of EdU signal showing cumulative progression of bidirectional replication forks after HU block release (dark grey, red, green) from core replication zones (light grey) (see methods). Merged replicate tracks are shown. **D**. Quantification of differential histone peptides detected in MSL-KO HME1 cells visualized as a volcano plot. Histone peptides with $|Log_2FC| \geq 0.5$ and FDR ≤ 0.05 relative to the control were considered differentially enriched. **E**. Heatmap showing Log_2 fold change relative to the mean value of control cells for the indicated modified histone peptides, and control cells treated with 50 nM Quisinostat (HDACi), used as a reference for substantial changes. Three biological replicates per condition are shown. Asterisks indicate significance by FDR.

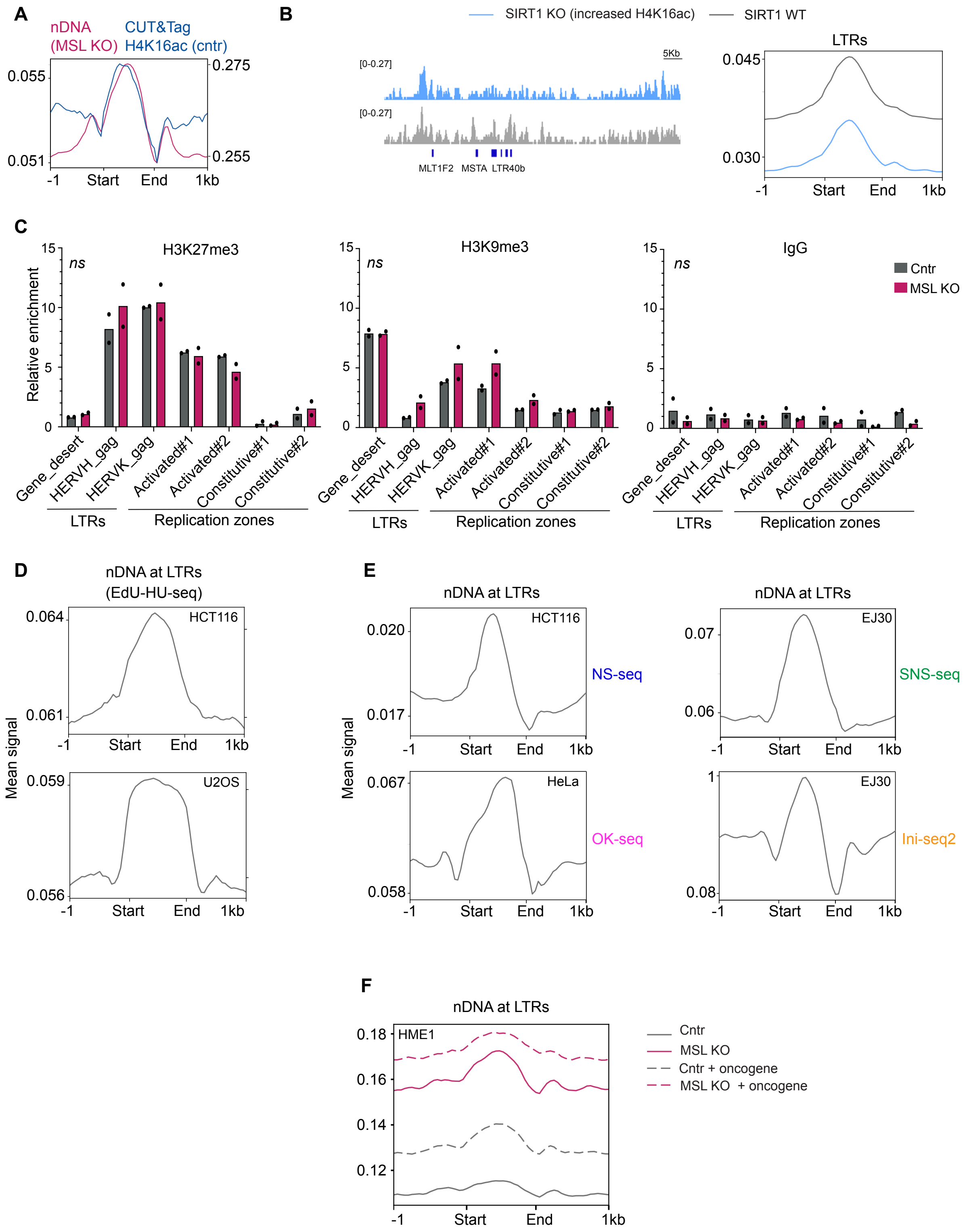

Figure S5

Figure S5: Nascent DNA profiles at LTRs.

A. Overlay of nDNA signal detected in MSL KO cells and CUT&Tag H4K16ac profiles in control cells at LTRs. **B**. Representative tracks (left) and metaprofiles (right) of nascent DNA signal detected by NS-seq at LTRs in the indicated conditions. A specific decrease of signal at LTR is observed. Datasets from GSE94403 (20). **C**. Quantification of repressive histone marks at the indicated regions by ChIP-qPCR. Mean and individual values of two biological replicates are plotted. Ns: non-significant (two-way ANOVA and multiple comparison testing assessing each element). **D**. Metaprofiles of EdU-HU signal at LTRs in the indicated cancer cells.

Datasets GSE212710 (48) and PRJNA397123 (61). Signals are stranded based on LTR orientation. **E**. nDNA metaprofiles at LTRs in the indicated cell lines. Datasets GSE94403 (20), PRJEB25180 (62) and GSE186675 (47). For Ini-seq2, normalized HL signal over LL signal, which sensitively detects replication origins (62), is plotted. **F**. Metaprofiles of EdU signal in the indicated HME1 cell lines at LTRs located in MSL KO-induced replication initiation sites. Signals are stranded based on LTR orientation.

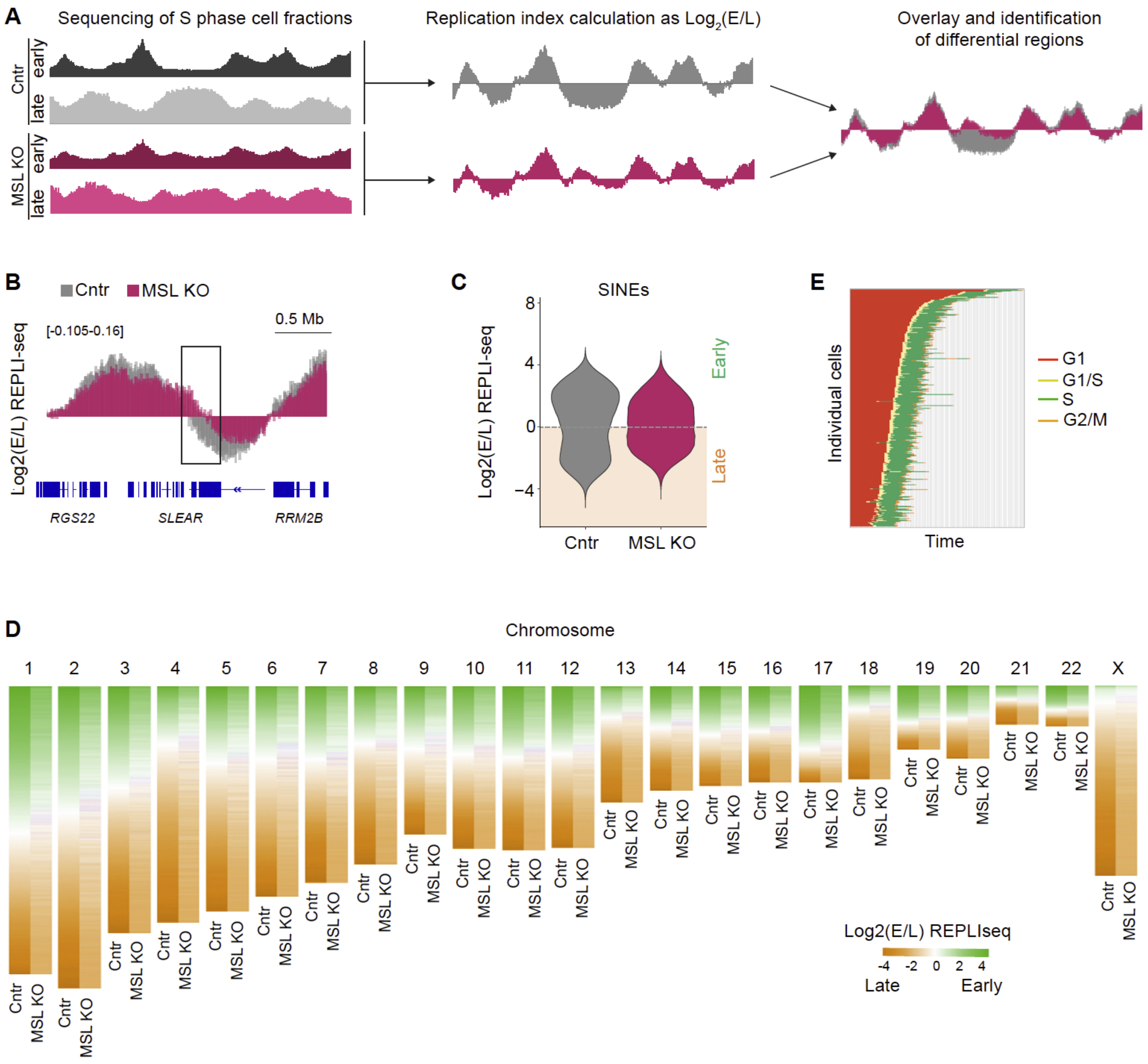

Figure S6: Global alterations in replication timing in the absence of H4K16ac

A. Schematic of REPLI-seq analysis and visualization steps. **B**. Tracks showing RT alterations in H4K16ac-depleted HME1 cells at the transition between early- and late-replicating domains. **C**. RT index of SINEs in the indicated cell lines. **D**. RT index attenuation in H4K16ac-depleted cells across all chromosomes. Each quantifiable 50kb bin of chromosomes is plotted. Bins are sorted by replication timing in control cells. **E**. Representative waterfall plot showing the duration of cell cycle phases in individual FUCCI(CA)2-expressing HME1 cells.
